# Phonon-driven intra-exciton Rabi oscillations in CsPbBr_3_ halide perovskites

**DOI:** 10.1038/s41467-023-36654-2

**Published:** 2023-02-24

**Authors:** Xuan Trung Nguyen, Katrin Winte, Daniel Timmer, Yevgeny Rakita, Davide Raffaele Ceratti, Sigalit Aharon, Muhammad Sufyan Ramzan, Caterina Cocchi, Michael Lorke, Frank Jahnke, David Cahen, Christoph Lienau, Antonietta De Sio

**Affiliations:** 1 Institut für Physik, Carl von Ossietzky Universität, 26129 Oldenburg, Germany; 2 Department of Molecular Chemistry & Materials Science, Weizmann Institute of Science, 76100 Rehovot, Israel; 3 Department of Materials Engineering, Ben-Gurion University of the Negev, 84105 Beer-Sheva, Israel; 4 Institut Photovoltaïque d’Île de France (IPVF), CNRS, Ecole Polytechnique, Palaiseau, 91120 France; 5 Sorbonne Université, CNRS, Collège de France, UMR 7574, Chimie de la Matière Condensée de Paris, Paris, F-75005 France; 6 Center for Nanoscale Dynamics (CeNaD), Carl von Ossietzky Universität, 26129 Oldenburg, Germany; 7 Institut für Theoretische Physik, Universität Bremen, 28359 Bremen, Germany; 8 Research Center Neurosensory Science, Carl von Ossietzky Universität, 26111 Oldenburg, Germany

**Keywords:** Condensed-matter physics, Materials for devices, Optical spectroscopy

## Abstract

Coupling electromagnetic radiation with matter, e.g., by resonant light fields in external optical cavities, is highly promising for tailoring the optoelectronic properties of functional materials on the nanoscale. Here, we demonstrate that even internal fields induced by coherent lattice motions can be used to control the transient excitonic optical response in CsPbBr_3_ halide perovskite crystals. Upon resonant photoexcitation, two-dimensional electronic spectroscopy reveals an excitonic peak structure oscillating persistently with a 100-fs period for up to ~2 ps which does not match the frequency of any phonon modes of the crystals. Only at later times, beyond 2 ps, two low-frequency phonons of the lead-bromide lattice dominate the dynamics. We rationalize these findings by an unusual exciton-phonon coupling inducing off-resonant 100-fs Rabi oscillations between 1s and 2p excitons driven by the low-frequency phonons. As such, prevailing models for the electron-phonon coupling in halide perovskites are insufficient to explain these results. We propose the coupling of characteristic low-frequency phonon fields to intra-excitonic transitions in halide perovskites as the key to control the anharmonic response of these materials in order to establish new routes for enhancing their optoelectronic properties.

## Abstract (web summary)

Coupling electromagnetic radiation with matter is promising to tailor optoelectronics properties of functional materials. Here, the authors demonstrate that internal fields induced by coherent lattice motions can be used to control transient excitonic optical response in halide perovskite crystals.

## Introduction

Coupling matter excitations, such as excitons, and localized electromagnetic radiation, e.g., via light modes in external optical or plasmonic resonators, may result in hybrid quasi-particles with both matter and field character^1^. Excitons dressed by a light mode may profoundly alter the energy landscape of the material and its quantum dynamics^1,2^. Such light-matter couplings are highly interesting for nanomaterial engineering, as they provide approaches for manipulating optoelectronic properties and photoinduced dynamics on the nanoscale, without modifying the material composition or crystal structure^3–9^. In the simplest view, the interaction of an exciton with a resonant quantized light mode is described by the Jaynes-Cummings model^1^. The coupling strength ℏΩ between the exciton and the field mode defines the energy oscillation rate between them. Strong coupling sets in when ℏΩ exceeds the damping rates of both uncoupled systems. In optical spectra, it manifests itself as peak splittings or sidebands to a bare exciton resonance^10^, and, in the dynamics, as Rabi oscillations^11^, a coherent, periodic energy exchange between matter and field.

Optical phonons in polar semiconductors are associated with electric fields confined inside the crystal and have a well-defined spectrum of allowed modes dictated by the specific crystal structure and material composition. The photoexcited charge density induces local displacements of the ions in the lattice generating a polarization field which can, in turn, interact with the charges. This leads to the formation of polaronic quasi-particles^12^ with enhanced mass^13^ and reduced mobility^12^ compared to free carriers. Usually, however, phonon fields are largely off-resonant with relevant electronic transitions. Hence, often, a single electronic state governs the interaction and the adiabatic treatment of polarons within the Born-Oppenheimer approximation is well justified. In contrast, strong light-matter coupling involves different electronic states.

There is increasing consensus that in halide perovskites (HaPs), the interaction of electronic excitations with the vibrational modes of the flexible polar lattice^14–16^ is crucial for their unique optoelectronic and transport properties^17–19^ that form the basis for their excellent device performance. Fundamental aspects of the underlying electron-phonon interactions in HaPs are, however, still debated. Established models developed for conventional semiconductors, such as the Fröhlich model^20^ describing long-range electron-phonon interactions, have mostly been used to explain the observations of time-resolved spectroscopic studies^14,21–25^. The Fröhlich model considers the lattice as a polarizable dielectric continuum and describes electron–phonon interactions by a coupling constant *α* which depends primarily on the values of the dielectric function in the high-frequency and static limit, on the charge density, and on a single phonon mode energy involved in the coupling^12,20^. Results from time-dependent optical Kerr effect spectroscopy^14^, transient THz photoconductivity^22^, two-photon photoemission dynamics^26^, and recent angle-resolved photoelectron spectroscopy experiments^25^ have been interpreted based on the formation of large electron or hole polarons on sub-ps timescales. Signatures of phonon coherences^23,24^ after impulsive optical excitation in ultrafast experiments have been rationalized within displacive excitation mechanisms, estimating electron-phonon couplings based on the Fröhlich model.

This model, however, neglects the real-space structure of the lattice and therefore cannot account for short-range interactions. Small polarons can be particularly important in low dimensional crystals, including two-dimensional HaPs^27^, and they have also been suggested to form in bulk HaPs^19^. So far, experiments have mostly been interpreted using an adiabatic treatment of the electron-phonon coupling, whereas atomistic simulations^28,29^ indicate that nonadiabatic couplings may also play an important role, raising the question whether the underlying Born-Oppenheimer approximation indeed suffices to explain the optoelectronic properties of HaPs. Recent studies also suggest more complex scenarios including important contributions of dynamic lattice disorder^30^ and anharmonicity^17,31,32^.

Here, using ultrafast two-dimensional electronic spectroscopy (2DES), combined with model simulations of the nonlinear optical response, we report a clear breakdown of the adiabatic exciton-phonon coupling regime in CsPbBr_3_ single crystals. Our data provide evidence for off-resonant Rabi oscillations between 1s and 2p excitons, driven by the characteristic low-frequency phonon modes of the lead-bromide lattice. This is in contrast with prevailing models for the electron-lattice interaction in HaPs. Our results suggest that the low-frequency phonon fields in the crystals can modulate the exciton population and thus may modify the transient optoelectronic properties of HaPs, analogous to strong light-matter couplings.

## Results

### Ultrafast two-dimensional electronic spectroscopy of CsPbBr_3_ crystals

**Fig. 1:**
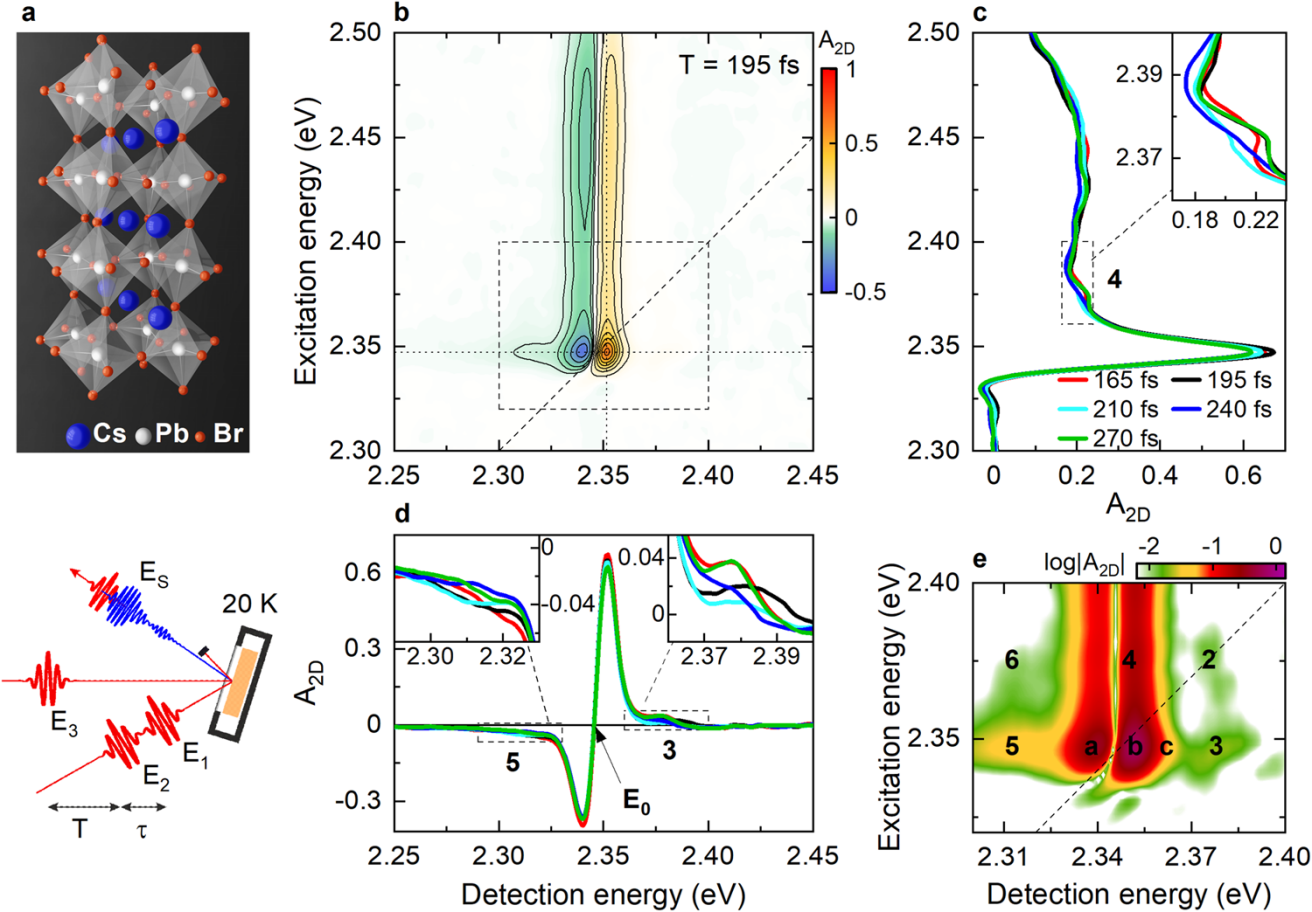
Reflection two-dimensional electronic spectroscopy (2DES) of CsPbBr_3_ single crystals at 20 K. **a** CsPbBr_3_ single crystals (ball-and-stick representation, upper panel) are resonantly excited and probed by a sequence of three ultrashort optical pulses (*E*_1_, *E*_2_, *E*_3_), time-delayed by the coherence time *τ* and the waiting time *T* (lower panel). **b** 2DES map at *T* = 195 fs showing a dispersive exciton diagonal peak at E_0_ = 2.345 eV and a vertically elongated free-carrier-induced cross-peak. **c**, **d** Cross-sections at a fixed (**c**) detection and (**d**) fixed excitation energy, indicated by the dotted lines in **b**, and for selected waiting times, reveal weak vibronic cross-peaks (3, 5) with a spacing of ~38 meV (310 cm^−1^) and amplitude oscillations mainly on the high-energy peaks (3, 4 in the insets in **d**, **c**, respectively). **e** A logarithmic scale plot of the exciton region highlights the subpeak structure (2–5).

We investigate CsPbBr_3_ single crystals in the orthorhombic phase at 20 K (Fig. 1a, upper panel). We perform 2DES measurements in a partially collinear configuration in reflection^33^ using a pair of phase-locked pump pulses, time-delayed by the coherence time τ, and a probe pulse, delayed by the waiting time T with respect to the second pump pulse (Fig. 1a lower panel).

For each T, we record differential reflectivity spectra as a function of τ and of the detection energy *E*_D_. The real part of the Fourier transform (FT) of this time domain signal along τ provides energy-energy 2DES maps A_2D_(*E*_X_, *T*, *E*_D_), with *E*_X_ being the excitation energy (Methods). Since we study optically thick bulk crystals in reflection, the measured A_2D_ signal is dominated by the real part of the nonlinear optical susceptibility of the crystals^33,34^, yielding dispersive lineshapes along *E*_D_ (Fig. 1b). The optical pulses allow for simultaneous resonant excitation of excitons and free carriers with an excess energy of ~200 meV in the continuum (Fig. S1). The 2DES data presented here have been recorded with a pump fluence of 4 µJ/cm^2^ ensuring low excitation densities below the Mott transition^33^ and orders of magnitude below the photodamage limit^35^.

The 2DES maps show a sharp dispersive diagonal peak at *E*_0_ = 2.345 eV, and a dispersive vertically elongated cross-peak at higher E_X_ (Fig. 1b). The diagonal peak is commonly assigned^33^ to 1s excitons with a binding energy of 40 meV^36^. A vertically elongated cross-peak appears upon excitation and its shape remains essentially unchanged up to 12 ps (Fig. S2). Its amplitude decays slowly on timescales of ~2.5 ps and >50 ps (Fig. S3). This cross-peak arises from the optical excitation of free carriers in the sample. Coulomb interactions between free-carrier excitations and excitons may induce a slight broadening of the exciton resonance (excitation-induced dephasing, EID) or a resonance shift (excitation-induced shift, EIS)^37^ and thus an optical nonlinearity that is detected at the exciton resonance. To check the effect of such many-body interactions on the 2DES lineshapes of our CsPbBr_3_ crystals, we have investigated the pump-fluence dependence of the 2DES maps (Fig. S4). The dispersive exciton diagonal peak and free-carrier-induced vertically elongated cross-peak slightly broaden along the *E*_D_ with increasing pump-fluence, consistent with EID, whereas no significant peak distortion, i.e., a transition in lineshape from dispersive to absorptive, is observed. Such a peak distortion could have reflected strong EIS^38^. Instead, the lineshapes remain dispersive over the investigated fluence range up to 20 µJ/cm^2^ (Fig. S4a–e). At this high fluence, however, the signal amplitude A_2D_ starts to saturate (Fig. S4f), a signature that the photoexcited carrier density approaches the Mott transition^33^. For the low fluence of 4 µJ/cm^2^, at which we conduct the experiments reported in the present manuscript, the exciton peak lineshape is dominated by bleaching and thus the dispersive shape of the peak primarily results from measuring the crystals in reflection^33^. Many-body interactions, specifically EID, contribute to the nonlinear spectrum as revealed by the presence of the vertically elongated cross-peak, but they are not the dominant nonlinearity for the exciton lineshape under our experimental conditions.

From the increase in cross-peak linewidth with excitation fluence (Fig. S4 and ref. ^33^), we deduce that EID dominates the cross-peak whereas EIS is weak under our experimental conditions. Similar EID-induced vertically elongated cross-peaks have been previously observed also in GaAs quantum wells^39^.

We now focus on the 1s exciton peak. Around its center, we detect a weak subpeak structure with cross-peaks spaced by ~38 meV (Fig. 1c–e, labeled 2–6). These cross-peaks are clearly pronounced on the higher energy side of the exciton diagonal peak, yet less well resolved on the lower energy side (Fig. 1c, d, insets). This asymmetry is evidenced in the cross-sections of the 2DES map along *E*_X_ (Fig. 1c) and *E*_D_ (Fig. 1d) taken at the exciton position. The absorption coefficient of similar CsPbBr_3_ single crystals was recently estimated using the optical constants obtained by spectroscopic ellipsometry^40^. At room temperature, this spectrum shows the excitonic 1s peak at ~2.41 eV, in agreement with our previous 2DES study^33^. In the room temperature data, however, no substructure can be seen due to the broad lineshapes.

**Fig. 2:**
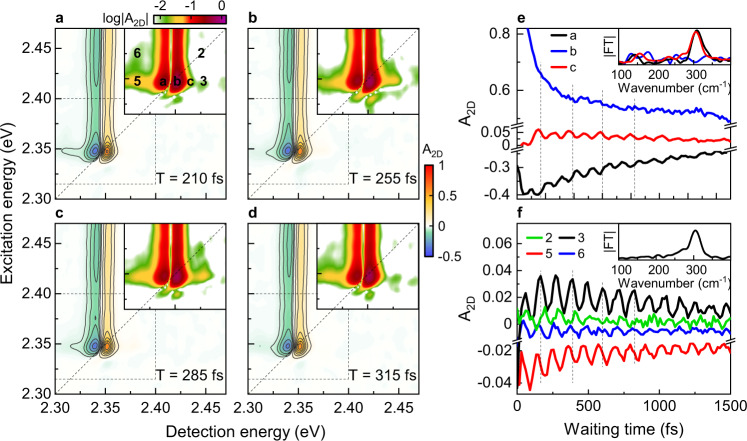
Waiting time dynamics of selected peaks in the 2DES maps reveal 310 cm^−1^ peak amplitude oscillations that are assigned to intra-exciton Rabi oscillations. **a**–**d** 2DES maps at selected waiting times T between 210 fs and 315 fs. A zoom into the exciton region on a logarithmic scale (insets) shows the oscillation of the subpeak structure. **e**, **f** Waiting time dynamics of selected peaks near the exciton resonance display amplitude oscillations of the A_2D_ signal with a period of 107 fs (310 cm^−1^), matching the spectral peak spacings (see also Fig. 1), but none of the phonon modes of these crystals. The insets show the Fourier transforms of selected dynamics.

2DES maps at selected waiting times are shown in Fig. 2a–d with logarithmic scale insets around the exciton region highlighting the subpeak structure. The waiting time dynamics of the A_2D_ signal at positions a and c, i.e., at the minimum (a) and on the high-energy side (c) of the dispersive 1s exciton peak, respectively, display amplitude oscillations with 107 fs period (310 cm^−1^ ≈ 38 meV) for *T* < 2 ps (Fig. 2e, black and red). The amplitude of these oscillations nearly vanishes at the maximum of the dispersive exciton resonance, *E*_0_ + δ (b, Fig. 2e blue), with *δ* = 4 meV. Pronounced amplitude oscillations at 310 cm^−1^ with an amplitude larger than 2% of the maximum A_2D_ signal are also observed at the subpeaks (Fig. 2f).

### Oscillatory dynamics of the excitonic peaks amplitude

**Fig. 3:**
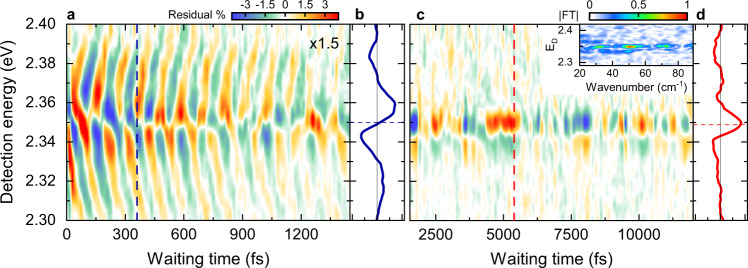
Residual map showing the oscillatory modulation of the A_2D_ signal averaged along the exciton region. **a**, **b** For T < 2 ps (**a**), the residuals show fast, 107 fs (310 cm^−1^) oscillations and (**b**) a dispersive lineshape centered around the maximum of the 2DES cross-section (cf. Figure 1d) at E_0_ + δ (dashed line in **b**). **c**, **d** For T > 2 ps (**c**), oscillations at ~30 cm^−1^ and 50 cm^−1^ (FT map in the inset. See also Figs. S5, 6) with (**d**) an absorptive lineshape centered around E_0_ (dashed line in **d**) are observed. This lineshape matches the predictions of a displaced harmonic oscillator model, showing that the oscillations reflect low-frequency coherent phonon wavepacket motion. The fast oscillations in (**a**) go beyond the displaced harmonic oscillator model and are assigned to intra-exciton Rabi oscillations.

To analyze the modulation of the exciton dynamics, we isolate the oscillatory part of the A_2D_ signal by averaging along *E*_X_ around the exciton spectral region (Fig. 1b, dashed square) and subtracting a slowly decaying bi-exponential background. The obtained residual map (Fig. 3) is dominated by the 310 cm^−1^ oscillations for *T* < 2 ps (Fig. 3a). At later times, two different, lower frequency components at ~30 cm^−1^ and 50 cm^−1^ are observed (Fig. 3c and Figs. S5, 6), corresponding to the Pb-Br-Pb bending and stretching modes of the HaP octahedra^14,17^. The spectral shape of the residual map along *E*_D_ is clearly different at early and later times. For *T* < 2 ps, we observe a dispersive-like shape of the residual map (Fig. 3b) centered around the maximum of the exciton signal at *E*_0_ + δ (dashed line). Its lineshape is very similar to the A_2D_ cross-section (Fig. 1d). At later times instead, the spectral dependence of the residual map for the low-frequency modes displays a symmetric, absorptive-like profile (Fig. 3d). This lineshape is consistent with that of conventional displaced harmonic oscillator (DHO) models^41^ for the coupling between the 1s exciton and the low-frequency phonon modes in the crystal (Fig. S8c).

Our 2DES data show distinct signatures of the coupling of excitons to persistent coherent phonons of the Pb-Br sublattice in CsPbBr_3_. In the Fröhlich model, the difference between the spatial charge density distribution of electron and hole forming the exciton^42^ determines the exciton-phonon coupling strength. This results in a finite dimensionless displacement Δ of the 1s exciton potential energy surface along the phonon coordinate with respect to the ground-state equilibrium configuration (Fig. S7a). Concurrently, it leads to a reduction of the 1s exciton energy by ${\varDelta }^{2}{E}_{V}=S{E}_{V}$, with ${E}_{V}$ being the phonon energy and *S* the Huang-Rhys factor^42^. In general, the exciton-phonon coupling manifests itself in characteristic phonon side-peaks around the exciton resonance in the optical spectra, provided that the phonon mode energy ${E}_{V}$ is large enough compared to the homogeneous linewidth defined by the exciton dephasing time ${T}_{2}$. Impulsive resonant optical excitation with optical pulses that are shorter in duration than the phonon period launches a coherent wavepacket onto the 1s exciton potential which oscillates in time with phonon frequency ${E}_{V}/\hslash$ (Fig. S7c). For the 30 and 50 cm^−1^ oscillations, ${E}_{V}$ is smaller than the homogeneous linewidth of the exciton resonance $2\hslash /{T}_{2}$. In this limit, the side-peaks are not resolved and the wavepacket motion results in a periodic modulation of the exciton resonance ${E}_{0}(T)={E}_{0}-{\varDelta }^{2}{E}_{V}[1-\,\cos ({E}_{V}T/\hslash )]$. In the 2DES maps, this leads to a small, periodic shift of the dispersive exciton lineshape along *E*_D_, with an energy shift that is much smaller than the resonance linewidth. In the residual maps, we plot the difference between the 2DES map at a given T and that averaged over one oscillation period. Therefore, the lineshape of the residuals is given by the derivative of the A_2D_(T) signal with respect to *E*_D_. This results in absorptive lineshapes of the residuals along *E*_D_ (Fig. S8c) matching those in Fig. 3c. The amplitude of the residuals oscillates with the phonon frequency, changing sign at every half period. This amplitude provides a good estimate of Δ and we deduce values between 0.1 and 0.2, thus small $S\le 0.04$, for the two phonon modes. Altogether, this confirms that the low-frequency oscillations in Fig. 3c reflect impulsive excitation of coherent phonon wavepackets induced by exciton-phonon coupling in our CsPbBr_3_ crystals.

For *T* < 2 ps instead, the dispersive shape of the residual map for the 310 cm^−1^ oscillations clearly does not fit to this model. If the 310 cm^−1^ oscillation was an optical phonon mode of the crystal, which we checked it is not, then ${E}_{V} > 2\hslash /{T}_{2}$ and the coherent wavepacket motion should give rise to distinct side-peaks in the nonlinear optical spectra (Fig. S8b) with amplitudes oscillating periodically in time. Such side-peak oscillations, however, could not explain the dispersive lineshape of the residuals observed in Fig. 3a (cf. Fig. S8d). Moreover, we note that the point labeled 5 in Fig. 1d, e is on the tail of the dispersive main exciton peak lineshape and is not a cross-peak. It oscillates nearly in phase with the oscillating main exciton peak, in contrast to expectations of a DHO model.

Dark excitons, arising from exciton fine structure splittings, have been reported slightly below the bright 1s exciton in HaPs^43^. The energy splittings between the bright and low-lying dark excitons in bulk HaPs are however of the order of only ~2 meV^43,44^, thus much smaller than the observed ~38 meV (310 cm^−1^) splittings between the main exciton and side-peaks in our 2DES maps. Moreover, the side-peaks in our 2DES maps are clearly on the high-energy side of the 1s exciton resonance. Therefore, we can rule out that lower-lying dark states from exciton fine structure splittings give rise to the observed spectral substructure.

Based on this analysis, the DHO model is unable to explain our observations (Fig. 3a). Weak resonances ~300–320 cm^−1^ observed in Raman studies^45–47^ have been tentatively assigned to an overtone of the 150 cm^−1^ mode, the highest frequency optical phonon in CsPbBr_3_^17^. In our 2DES data we do not detect any significant amplitude oscillation at 150 cm^−1^ nor any phonon side-peaks associated with this frequency. Moreover, control measurements of layered 2D-HaPs, namely (BA)_2_(MA)_n-1_Pb_n_I_3n+1_ with *n* = 3 and BA = CH_3_(CH_2_)_3_NH_3_ as the organic spacer, having similar low-frequency phonon modes as CsPbBr_3_, but substantially larger exciton binding energy^48^, show neither 310 cm^−1^ oscillations nor dispersive shape of the residual map, but only low-frequency lead-halide phonon oscillations with residuals matching the DHO (Fig. S9b). As such, we exclude that the observed 310 cm^−1^ oscillation in our CsPbBr_3_ data arises from a phonon overtone.

Temporal oscillations with frequencies higher than coherent phonons have been recently observed in CsPbBr_3_ for excitation energies below the optical bandgap in 2D-optical Kerr effect experiments in transmission^49^. Those oscillations were attributed to birefringence arising from an instantaneous, off-resonant nonlinear optical response induced by the pump pulses^49,50^. In contrast, in our 2DES experiments, the high-frequency 310 cm^−1^ oscillations arise from a resonant nonlinearity of the crystals and appear only for excitation and detection energies around the exciton resonance. Potential contributions of birefringence-induced below-bandgap oscillations as discussed in ref. ^49^ are not relevant under our experimental conditions.

THz-pump-VIS-probe studies of MAPbI_3_ thin films observed a transient shift of the optical bandgap and temporal oscillations in the differential transmission amplitude resulting from the coupling of the Pb-I bending mode at 1 THz to the crystal bandgap^15^. In contrast to our 2DES experiment, the THz pump resonantly and selectively excites low-frequency phonon modes, but it does not excite electronic populations, i.e., excitons or free carriers. It instead induces phonon coherences in the crystal ground state. This results in an oscillatory, strain-induced change in the bandgap of the material that is probed by the time-delayed visible probe pulse. In contrast, here we do not selectively excite specific phonon modes of our CsPbBr_3_ crystals and hence the observed low-frequency phonon wavepacket motion (Fig. 3c and S5d) reveals the Pb-Br modes that most strongly couple to the exciton. Most importantly, within the first ~2 ps, we observe oscillations with much higher frequencies (Figs. 2e, f and 3a) than those reported in ref. ^15^.

All this raises questions about the origin of the observed 310 cm^−1^ amplitude oscillations. Exciton resonance energy oscillations with 310 cm^−1^ frequency would still result in absorptive-like spectral shape of the residuals and thus cannot account for the observed dispersive shape in Fig. 3a. The latter may be caused by a change either in the exciton population or in the exciton transition dipole moment^51^. Changes in the exciton transition dipole moment, however, will not give rise to side-peaks in the optical spectra. Here, we propose that the low-frequency phonon modes in the CsPbBr_3_ not only couple to the 1s exciton, but also drive dipole-allowed transitions to higher-lying exciton states resulting in coherent oscillations of the exciton population. These oscillations modulate the exciton peak amplitude in 2DES leading to the dispersive shape of the residuals.

## Discussion

Within the three-dimensional exciton hydrogen-like progression commonly assumed for bulk HaPs^43^, the lowest-lying resonance is a 1s exciton carrying the largest oscillator strength, whereas higher-lying s-states are much weaker. Excited-state transitions between s-states are dipole forbidden and thus signatures of higher-lying s-states could only appear in weak diagonal peaks, but cannot explain the cross-peaks nor their oscillations in our 2DES data. Within such a hydrogen-like model, transitions between the 1s exciton and the higher-lying optically dark 2p exciton are dipole allowed. They may be driven by external THz fields^52,53^ or, as we argue here, by the internal electric field associated with the low-frequency phonons inside the crystals. In CsPbBr_3_, where the exciton binding energy for the lowest energy exciton (1s) is 40 meV^36^, the energy splitting between 1s and 2p is $\varDelta {E}_{1s,2p}\approx 30$ meV. Thus, in principle, the low-frequency phonon fields can off-resonantly drive transitions between them. This implies that they can induce a periodic transfer of a small fraction of the 1s exciton population to the 2p exciton state and back. Consequently, population oscillations between the exciton states transiently modulate the excitonic optical absorption of the crystal at a frequency governed by the splitting between the coupled states. In the nonlinear optical spectra, this will result in side-peaks of the 1s exciton and peak amplitude oscillations with frequencies determined by the population oscillations. The coupling strength inducing such splitting is governed by the dipole coupling $\hslash \varOmega \approx {\mu }_{1s,2p}{E}_{ph}$, with ${\mu }_{1s,2p}$ being the amplitude of the 1s-2p-transition dipole moment and ${E}_{ph}$ the phonon field amplitude. An estimation of ${\mu }_{1s,2p}$ based on the overlap integral of hydrogenic 1s and 2p wavefunctions, and of ${E}_{ph}$ results in a coupling strength of $\hslash \varOmega \approx 8$ meV, a factor of two larger than the value deduced from our experiments (Supplementary Note 2, 2.2.1). This is ~27% of the bare 1s-2p energy splitting $\varDelta {E}_{1s,2p}$, suggesting that this exciton-phonon field dipole coupling in CsPbBr_3_ crystals can reach the ultrastrong coupling limit, in analogy to light-matter couplings^54^.

Since $\varDelta {E}_{1s,2p}\approx 5{E}_{V}$, the phonon field drives the 1s-2p transition off-resonantly. As such, the rotating wave approximation (RWA), usually adopted in the Jaynes-Cummings model, is not justified, and the full exciton-phonon field interaction has to be considered. This includes both RWA terms and counter-rotating (CR) terms of the interaction (Supplementary Note 2, 2.2.2). The RWA terms promote energy-conserving transitions between $|1s,\nu+1\rangle$ and $|2p,\nu \rangle$, where $|n,\nu \rangle$ are vibronic states in a non-displaced basis with $n=1s,2p$ and $\nu=0,1,2,{{{{\mathrm{..}}}}}.$ being the vibrational quantum number. The CR terms induce transitions between $|1s,\nu \rangle$ and $|2p,\nu+1\rangle$ vibronic states and thus promote the excitonic transitions from the 1s to 2p manifold (or 2p to 1s) while concurrently creating (or destroying) a phonon quantum. We argue that here those CR terms even dominate the coupling and result in the observed 310 cm^−1^ oscillations.

**Fig. 4:**
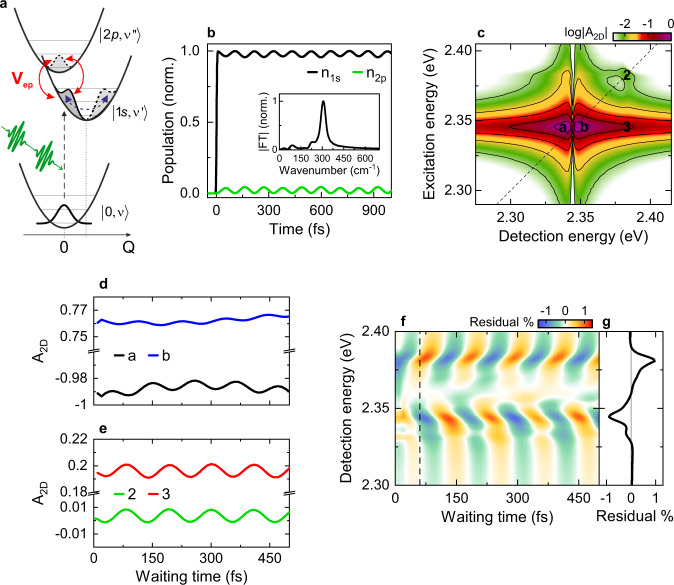
Model simulations of phonon-driven intra-exciton Rabi oscillations in CsPbBr_3_ crystals. **a** Displaced harmonic oscillator model including the bright $|1s,\nu {\prime} \rangle$ and dark $|2p,\nu {\prime\prime} \rangle$ exciton states, with $\nu {\prime},\nu {\prime\prime}=0,1,2,{{{{\mathrm{..}}}}}.$, the vibrational quantum number associated with the 50 cm^−1^ phonon mode. We include the dipole coupling between 1s and 2p via a quantized phonon field driving Rabi oscillations between them. The coupling V_ep_ is governed by the 1s–2p transition dipole moment and by the phonon field. **b** Simulated dynamics of the electronic 1s (black) and 2p (green) populations n_1s_ and n_2p_. The dynamics of those populations show Rabi oscillations with a dominant period of ~109 fs (307 cm^−1^), as seen by taking the Fourier transform of n_1s_ (inset). **c** An exemplary simulated 2DES map at *T* = 80 fs (logarithmic scale plot) reveals high-energy side-peaks (2, 3) with spacings of ~38 meV (307 cm^−1^) arising from the mixing of 1s and 2p vibronic states induced by the phonon mode. **d**, **e** In the waiting time dynamics, this coupling leads to peak amplitude oscillations with a period of ~109 fs and with similar distribution as the amplitude modulation in the experiment. **f**, **g** The residual map shows a dispersive-like spectral profile along the detection energy axis, in good agreement with that observed in the experiments.

To support this argument, we simulate the exciton optical response of the CsPbBr_3_ crystals using a three-state DHO model (Fig. 4a) including the crystal ground-state, the 1s and 2p excitons, and the dipole coupling between them via a quantized 50 cm^−1^ (${E}_{V}=6.3$ meV) phonon field based on the full exciton-phonon field interaction. In this simplified picture (Fig. 4a), we restrict our model to excitons at $k=0$ in momentum space and neglect higher k-states. This approximation is reasonably well justified since our experimental data show essentially no disorder-induced inhomogeneous broadening. Thus, the optical pulses can only couple to excitons with wavevector $k\approx 0$, whereas transitions to optically dark higher k-states excitons are negligible to a first approximation. In this model, the impulsive optical excitation launches coherent phonon wavepackets oscillating with 50 cm^−1^ frequency on the optically bright 1s exciton potential energy surface. The phonon field drives the dipole-allowed transition between 1s and 2p with ${V}_{ep}=\hslash \varOmega \sqrt{\nu+1}$ (Supplementary Note 2, 2.2.2). We find good agreement between simulation and experiment for $\hslash \varOmega=3.7$ meV. The coupling induces hybridization of $|1s,\nu \rangle$ and $|2p,\nu \rangle$ vibronic states which, upon optical excitation of the 1s exciton, leads to Rabi oscillations between the populations, n_1s_ and n_2p_, of 1 s and 2p excitons (Fig. 4b). Since the displacement of the 1s exciton potential energy surface is small, the optical excitation mainly populates $|1s,0\rangle$, and weakly $|1s,1\rangle$, whereas the population of higher-lying $|1s,\nu > 1\rangle$ states is negligible (Fig. S7b). RWA interaction terms cannot induce couplings between $|1s,0\rangle$ and the 2p manifold (Figs. S10 and S11c) and thus they cannot give rise to the oscillations of n_1s_ and n_2p_ in Fig. 4b. The dominant frequency (307 cm^−1^, Fig. 4b inset) of these oscillations arises from the energy splitting between hybridized 1s and 2p vibronic states involving $|1s,0\rangle$. In the range of $\hslash \varOmega$ considered here, the lowest-lying 1s-2p-phonon eigenstate can be approximated as arising from the hybridization of mainly $|1s,0\rangle$ and $|2p,1\rangle$ with the contributions of other higher-lying 1s and 2p vibronic states being negligible. Therefore, the dominant contribution to the splitting, and hence to the population oscillations, arises from the CR-induced coupling between $|1s,0\rangle$ and $|2p,1\rangle$ (Fig. S12). Thus CR terms of the interaction are necessary to explain our data.

In the 2DES maps, this coupling results in diagonal peaks at the energies of the bright eigenstates and their cross-peaks. The strongest diagonal peak marks the lowest-lying bright eigenstate which mainly arises from the mixing of $|1s,0\rangle$ and $|2p,1\rangle$ and it is approximately at the energy of the ground-state-to-$|1s,0\rangle$ transition. This is the main diagonal exciton peak at 2.345 eV Fig. 4c and Fig. 1e. The other, weaker diagonal peak arises from the brightening of the ground-state-to-$|2p,1\rangle$ transition upon coupling and gives rise to the weak diagonal peak at position 2 in the 2DES maps (Figs. 4c and  1e). As such, in the 2DES maps we observe a main diagonal peak at approximately the energy of the 1s exciton transition, a second much weaker diagonal peak approximately around the $|2p,1\rangle$ transition, and their cross peaks, in agreement with our experimental observations.

Population oscillations result in a periodic modulation of the nonlinear optical polarization, which leads to weak vibronic side-peaks on the high-energy side of the exciton resonance in the 2DES maps (Fig. 4c and Fig. S13b). The spacing between the side-peak and the main peak reflect the energy splittings discussed above. In the dynamics, ~109-fs peak amplitude oscillations (Fig. 4d, e) appear, matching the peak spacings. The resulting residual map (Fig. 4f) shows a dispersive-like spectral profile with a sign change around the maximum of the exciton lineshape (Fig. 4g and Fig. S13), in agreement with the experiments. We note that the position at which the oscillations are most strongly reduced is not at the zero-crossing of the dispersive exciton resonance, but around its maximum in both simulation and experiment (indicated as peak b in Figs. 4c and 1d, e). This is because the Rabi oscillations do not occur between two purely electronic states but involve higher-lying vibronic 1s and 2p manifolds. Importantly, the frequency of these phonon-induced intra-exciton Rabi oscillations (Fig. 4b) is not simply a multiple of the phonon frequency, but reflects the energy splitting between the coupled $|1s,0\rangle$ and $|2p,1\rangle$ states, i.e., hybridized 1s-2p-phonon modes induced by CR terms. This is fundamentally different from the Fröhlich coupling. For a coupling strength $\hslash \varOmega=3.7$ meV, $\hslash \varOmega /\varDelta {E}_{1s,2p}=0.12$ which is on the onset of the ultrastrong coupling regime^54^. Our results thus imply that the quantum nature of the phonon fields in HaPs may play an important role for this exciton-phonon coupling in analogy to light-matter couplings^1,54,55^.

In our model we assume that only the 50 cm^−1^ mode is responsible for the coupling. This is a reasonable approximation since the detuning between the mode energy ${E}_{V}$ and $\varDelta {E}_{1s,2p}$ is smaller than for the 30 cm^−1^ mode. From our 2DES data we estimate similar Huang-Rhys factors for both phonon modes. Thus, a larger coupling strength for the 30 cm^−1^ mode is needed to compete with Rabi oscillations induced by the 50 cm^−1^ mode. As such, while we cannot strictly rule out that also the 30 cm^−1^ phonon mode is involved in the coupling, we expect that the 50 cm^−1^ mode dominates.

The phonon-induced electric fields in the crystals can promote off-resonant Rabi oscillations between $|1s,\nu \rangle$ and $|2p,\nu \rangle$ vibronic states only as long as the coherence between them is maintained. Thus, the dephasing between those vibronic states determines the dephasing of the Rabi oscillations. Since the phonon dephasing (~7–8 ps at 20 K) is much longer than the timescale over which we observe Rabi oscillations (~2 ps), our results suggest that the decay of the Rabi oscillations can be taken as a measure of the dephasing time between 1s and 2p excitonic manifolds. As such, the revealed Rabi oscillations may offer a way to study the dephasing mechanisms within the excited exciton manifold which is important, e.g., for applications in light-emission.

Our results also suggest that for materials with similar low-frequency phonon spectra as CsPbBr_3_, the exciton binding energy can be used to control the Rabi oscillations dynamics and thus the transient excitonic response. Specifically, larger exciton binding energies in the presence of a similar phonon spectrum result in larger 1s-2p splitting and thus larger detuning with the phonon mode energy. Based on our model, we thus expect no significant Rabi oscillations in this scenario. This is confirmed by the results of control measurements on layered 2D-HaPs (Fig. S9) having an exciton binding energy more than four times larger^48^ compared to bulk CsPbBr_3_ but a similar low-frequency phonon spectrum of the lead-halide lattice^56^.

In conclusion, we have shown that in CsPbBr_3_ single crystals, internal electric fields generated by low-frequency phonon modes induce mixing of 1s and 2p excitons leading to Rabi oscillations for up to ~2 ps at 20 K. These intra-exciton Rabi oscillations cannot be explained by conventional models for electron-phonon couplings in the adiabatic regime. The disclosed mechanism can crucially contribute to explain the strongly anharmonic response of HaPs and, in relation to that, the plethora of peculiar behaviors exhibited by these materials^57,58^. Importantly, the reported observations call for the development of new theoretical approaches beyond the status quo models originally developed for conventional semiconductors, which in spite of their applicability to HaPs as demonstrated also in this work, still fundamentally rely on the Born-Oppenheimer approximation. Only in this way, it will be possible to fully unfold the behavior of this emerging class of materials not only as candidates for next-generation optoelectronic devices, but also as platforms to study light-matter phenomena in the strong and ultrastrong coupling regime.

## Methods

### CsPbBr_3_ crystal synthesis and characterization

Powders of cesium bromide (CsBr) (Aldrich, 99.999%) without further purification and lead-bromide (PbBr_2_) (Aldrich, ≥98%) that was dried in a vacuum oven overnight, were dissolved in dimethylsulfoxide (DMSO) (Aldrich, ≥99.9%). Acetonitrile (MeCN) (BioLab LTD, HPLC grade, 99.97%) and methanol (MeOH) (BioLab LTD, HPLC grade, 99.95%) were used as received. A 0.45 M solution (slightly below the ~0.5 M solubility limit) of the perovskite precursors (equimolar amounts of CsBr and PbBr_2_) in the same solution of DMSO was prepared in ambient air (ca. 45% RH) under continuous stirring at ~50 °C, until no powder is observed. It is important to mix the precursors in the same volume, because the room temperature solubility limit of CsBr in DMSO is ca. 0.25 M when it is dissolved separately. After cooling to room temperature, the DMSO solution was titrated (dropwise under continuous stirring) with MeCN. During the titration, a yellow-orange precipitant appeared with addition of each drop and quickly re-dissolved. As the system gets closer to the saturation point (more pronounced when MeCN is added), a permanent white solid precipitates. At MeCN: DMSO ratios of 1.1:1 the yellow-orange precipitate no longer re-dissolved. These saturated solutions were thoroughly sealed (to prevent loss of the volatile MeCN) and stirred for 24 hours at 50 °C. After heating for 24 h, a pale green-yellow strongly-fluorescent precipitant was clearly observed along-side the other precipitated species. The saturated solutions can be further stored (best in the dark) for at least several months, until their use for crystal growth. Before crystal growth, the saturated precursor solutions are filtered with PTFE 0.2 μm pore-size syringe filters. No noticeable differences in crystal growth or kinetics were observed between properly stored or freshly prepared solutions.

The filtered precursor solutions were placed in a clean crystallization flask and covered with a filter paper and a glass petri-dish on top to limit anti-solvent vapor diffusion. The covered crystallization flask was then placed inside a deeper, flat-bottomed, glass dish, which contained MeCN (anti-solvent). The setup was left at ambient conditions in a quite location for further growth. After ~2–3 days the crystals were taken out. Typical single crystals were collected from the crystallization solution. The crystallographic orientations are verified via XRD analysis in a specular reflection mode, where the (101) plane is set as the reflection plane. For further details see ref. ^59^.

### Two-dimensional electronic spectroscopy

We perform low-temperature two-dimensional electronic spectroscopy (2DES) in reflection using a home-built setup in the partially collinear geometry as detailed in ref. ^33^. Briefly, the broadband optical pulses, with a spectrum ranging from ~2.00 eV to ~2.55 eV (Fig. S1), are generated by a home-built non-collinear optical parametric amplifier (NOPA), pumped by the second-harmonic of a regeneratively amplified Ti:Sapphire laser (Spectra Physics Spitfire Pro, 5 kHz repetition rate). Chirped mirrors (Laser Quantum DCM9) are used to pre-compress the pulses. The phase-locked pump pulse pair used for the excitation is generated by a TWINS^60,61^ interferometer consisting of α-BBO wedges. The dispersion introduced by the wedges is compensated by an additional pair of chirped mirrors (Laser Quantum DCM9) introduced in the pump arm of the setup. To scan the coherence time τ (i.e., the time delay between the two pump pulses) and the waiting time *T* (time delay between the second pump and probe pulse), we use motorized delay stages (Physik Instrumente M112.1DG, M413.2DG). During each measurement, we monitor *τ* by recording the autocorrelation of the two pump pulses with a photodiode before the sample. Pump and probe beams are focused onto the sample through the quartz window of a cryostat (KONTI Micro, CryoVac), each to a round spotsize of ~120 µm in diameter, using spherical mirrors before the cryostat. The beam area is carefully characterized with a beam camera (Thorlabs DCC1545M-GL). During the measurements, the sample is kept at 20 K in the cryostat. The probe beam reflected from the sample is dispersed into a monochromator and collected with a high-speed CCD line camera (e2v AviiVa EM4).

The measured differential reflection from the sample as a function of *τ* and *T* and of the detection energy *E*_D_ is given by $\frac{\varDelta R}{R}(\tau,T,{E}_{D})=\frac{{R}_{on}(\tau,T,{E}_{D}) \, - \, {R}_{off}({E}_{D})}{{R}_{off}({E}_{D})}$, with ${R}_{on}$ and ${R}_{off}$ the reflected probe spectra with the pump on and off, respectively. By taking the real part of the Fourier transform of the measured $\varDelta R/R$ signal along *τ*, we obtain the 2DES energy-energy maps ${A}_{2D}({E}_{X},T,{E}_{D})$ at each *T* as a function of *E*_D_ and of the excitation energy *E*_X_. To characterize the time resolution of the setup, we record second-harmonic generation frequency-resolved optical gating (SHG-FROG) maps of the cross-correlation between pump and probe arms outside the cryostat introducing an additional quartz window into the beam path to account for the window of the cryostat. This allows us to compensate the chirp introduced by the window of the cryostat with chirp mirrors before the setup. The recorded SHG-FROG map after chirp compensation indicates a time resolution of ~13 fs (Fig. S1). For all measurements shown in the main manuscript, the excitation fluence is set to 4µJ/cm^2^.

## Supplementary information

Supplementary Information

## Data Availability

The data that support the findings of this study are available from the authors upon request.
