# Supplementary material for: Phonon-driven intra-exciton Rabi oscillations in CsPbBr3 halide perovskites
Source: Nat Commun. 2023 Feb 24;14:1047. doi: 10.1038/s41467-023-36654-2 (PMC9958027; doi:10.1038/s41467-023-36654-2)
Supplement: Supplementary file 1 — Supplementary Information [file 41467_2023_36654_MOESM1_ESM.pdf]

Supplementary Information for

Phonon-driven Intra-exciton Rabi Oscillations in $CsPbBr_3$ Halide Perovskites

Xuan Trung Nguyen[1], Katrin Winte[1], Daniel Timmer[1], Yevgeny Rakita[2,3], Davide Raffaele Ceratti[2,4], Sigalit Aharon[2], Muhammad Sufyan Ramzan[1], Caterina Cocchi[1,5], Michael Lorke[6], Frank Jahnke[6], David Cahen[2], Christoph Lienau[1,5,7], Antonietta De Sio[1,5*]

1 Institut für Physik, Carl von Ossietzky Universität, 26129 Oldenburg, Germany.

2 Department of Molecular Chemistry & Materials Science, Weizmann Institute of Science, 76100 Rehovot, Israel.

3 Department of Materials Engineering, Ben-Gurion University of the Negev, 84105 Beer-Sheva, Israel.

4 Institut Photovoltaïque d'Île de France (IPVF), CNRS, Ecole Polytechnique, Palaiseau, 91120, France and Sorbonne Université, CNRS, Collège de France, UMR 7574, Chimie de la Matière Condensée de Paris, Paris F-75005, France.

5 Center for Nanoscale Dynamics (CeNaD), Carl von Ossietzky Universität, 26129 Oldenburg, Germany.

6 Institut für Theoretische Physik, Universität Bremen, 28359 Bremen, Germany.

7 Research Center Neurosensory Science, Carl von Ossietzky Universität, 26111 Oldenburg, Germany.

*Correspondence to: antonietta.de.sio@uni-oldenburg.de

Supplementary Notes

Supplementary Note 1. First-principles simulations of the electronic and vibrational properties of $CsPbBr_3$.

We performed first-principles simulations, based on density-functional theory (DFT) and many-body perturbation theory, to inspect the electronic, optical, and vibrational properties of $CsPbBr_3$. We found that the highest-occupied band has hybridized Pb-s and Br-p character, while the lowest-unoccupied one has Pb-p character. The optical transitions stemming from these two states are evidently optically allowed and contribute from different k-points in the Brillouin zone to the lowest-energy excitons, which are assigned 1s and 2s character according to the H-like series. The contributions to these excitons coming from two states only (one occupied and one unoccupied) enforce the assumptions behind Elliot's model. The subsequent excitons with 2p character, although stemming again from the highest valence band to the lowest conduction band, are optically forbidden due to destructive interference between the single particle transitions.

Calculations of the phonon energies for pristine $CsPbBr_3$ indicate that the highest phonon energy is at values of 18.5 meV or 149 cm^{-1} , which is comparable with values reported in the literature[1,2]. These values remained unchanged upon electron and hole doping of the crystal. The calculations were performed using DFT as implemented in the Vienna ab-initio simulation package (VASP)[3,4] with the projected augmented wave method[5,6]. The atomic and electronic structures were determined via the PBE functional. A plane wave basis with an energy cutoff of $E_{cut} = 500\,\mathrm{eV}$ and a $(8 \times 8 \times 8)$ Monkhorst-Pack **k**-point sampling has been used for calculations of the primitive unit cell. Phononic properties have been calculated on the basis of density-functional perturbation theory[7] using a (5x5x5) supercell, containing 625 atoms.

Supplementary Note 2. Model simulations of the exciton dynamics and 2DES spectra.

2.1 Displaced harmonic oscillator model. The coupling of 1s excitons to optical phonons in a disorder-free bulk semiconductor crystal can be in general described by the Hamiltonian[8]

$$H_0 = \sum_{\vec{k}} \hbar\omega_{1s,\vec{k}} c^\dagger_{1s,\vec{k}} c_{1s,\vec{k}} + \sum_{\vec{w}} \hbar\omega_v b^\dagger_{\vec{w}} b_{\vec{w}} + \sum_{\vec{w},\vec{k}} V_{1s}\left(\vec{w}\right) c^\dagger_{1s,\left(\vec{k}+\vec{w}\right)} c_{1s,\vec{k}} \left(b_{\vec{w}} + b^\dagger_{\vec{w}}\right) \tag{1}$$

with the first term indicating the momentum-dependent energy $\hbar\omega_{1s,\vec{k}}$ of the lowest-lying exciton electronic state $|1s\rangle$ as a function of the wavevector $\vec{k}$. The energy of the crystal electronic ground state $|0\rangle$ is taken as a reference and set to zero. The creation $c^\dagger_{1s,\vec{k}}$ (annihilation

$c_{1s,\vec{k}}$) operators create (destroy) the 1s exciton with wavevector $\vec{k}$. The second term on the right-hand-side of equation (1) describes the phonon ladder for the phonon mode with energy $\hbar\omega_v$, and with $b^\dagger_{\vec{w}}$ and $b_{\vec{w}}$ being the creation and annihilation operators for the phonon with wavevector $\vec{w}$, respectively. The third term accounts for the exciton-phonon coupling between 1s excitons and the phonon mode with coupling matrix elements $V_{1s}\left(\vec{w}\right)$. The physical origin of these coupling matrix elements can be either that of a lattice continuum model describing long-range interaction, as in the Fröhlich model, or may result from short-range interactions, as in a Holstein model[9]. In bulk halide perovskites, it has been suggested that both long- and short-range electron-(exciton-)phonon interactions may be involved in the optoelectronic properties, with the lead-halide stretching and bending modes being dominantly involved in the coupling to electronic excitations[10,11]. In both cases, the exciton-phonon coupling leads to characteristic phonon sidebands to the 1s electronic resonance in the optical spectra. The intensity of those sidebands is determined by the coupling matrix elements via the Huang-Rhys factor S [8]. The low frequency amplitude oscillations persisting beyond 2 ps observed in the experimental data (Fig. 3c of the manuscript) can be well described within the framework of a simplified version of equation (1), a displaced harmonic oscillator (DHO) model[8]. Thus, we restrict the simulations to the coupling of one 1s exciton to a single, harmonic low frequency phonon mode. We consider vibronic states $\left|n,v\right\rangle$, with $n=0,1s$ indicating the electronic state and $v=0,1,2,...$ the vibrational quantum number for each electronic state. In a non-displaced vibronic basis, the DHO Hamiltonian is expressed as

$$H_0=\hbar\omega_v b^\dagger b+\left[\hbar\omega_{1s}+\hbar\omega_v\Delta_{1s}\left(b^\dagger+b\right)+\hbar\omega_v\Delta_{1s}^2\right]c^\dagger_{1s}c_{1s} \quad (2)$$

where the first term on the right-hand-side describes the phonon ladder with $\hbar\omega_v$ the phonon energy. The second term in equation (2) accounts for the coupling of the 1s exciton to the phonon mode. The minimal energy of the exciton state is given by the sum of the electronic energy $\hbar\omega_{1s}$ and the reorganization energy $\hbar\omega_v\Delta_{1s}^2$ induced by the exciton-phonon coupling via the dimensionless displacement $\Delta_{1s}=\sqrt{S}$. The latter describes the real space shift of the 1s exciton potential energy surface along the dimensionless phonon coordinate Q with respect to the ground state equilibrium configuration (Fig. S7a) induced by the exciton-phonon coupling. The exciton-phonon coupling strength $\hbar g=-\Delta_{1s}\hbar\omega_v$ is also expressed in terms of Δ_{1s}. From the experimental residual maps shown in Fig. 3c of the main manuscript, we estimate displacements of Δ_{1s} ~0.1-0.2 for the 30 and 50 cm^{-1} frequency modes. As discussed in the

manuscript, this single exciton DHO model (Figs. S7-8) nicely explains the absorptive-like shape of the residual map at later waiting times T>2ps (Fig. 3c). It cannot account, however, neither for the dispersive-like shape of the residuals in Fig. 3b nor for the subpeak structure around the exciton peak in the 2DES maps shown in Figs. 1 and 2.

2.2 Three-state DHO model with phonon-induced 1s-2p coupling. To model the 2DES spectra and dynamics at earlier times, we extend the DHO model to include the optically dark 2p exciton state, and the dipole coupling between 1s and 2p excitons mediated by the phonon mode. In this model, the vibronic basis states are $\left|n,v\right\rangle$, with $n=0,1s,2p$. The Hamiltonian describing the free evolution of this system, expressed again in a non-displaced vibronic basis, can be written as

$$\begin{aligned} H_0 &= \hbar\omega_v b^\dagger b + \left[\hbar\omega_{1s} + \hbar\omega_v\Delta_{1s}\left(b^\dagger + b\right) + \hbar\omega_v\Delta_{1s}^2\right]c_{1s}^\dagger c_{1s} + \\ &+ \hbar\omega_{2p}c_{2p}^\dagger c_{2p} + \hbar\Omega\left(b^\dagger + b\right)\left(c_{1s}^\dagger c_{2p} + c_{2p}^\dagger c_{1s}\right) \end{aligned} \quad (3)$$

where the first and second terms on the right-hand-side describe the phonon ladder, with phonon energy $\hbar\omega_v = E_V = 6.3\,\text{meV}$ (~50 cm^{-1}), and the coupling of the 1s exciton to the phonon mode as in equation (2). In all simulations we take Δ_{1s} = 0.1. The third term in (3) describes the 2p exciton energy $\hbar\omega_{2p} = \hbar\omega_{1s} + \Delta E_{1s,2p}$. The energy splitting $\Delta E_{1s,2p} = 30\,\text{meV}$ is determined by the exciton binding energy of 40 meV for $CsPbBr_3$ crystals[12]. For simplicity, we have assumed that the displacement for the 2p potential energy surface is negligible and set it to zero $\Delta_{2p} = 0$ since, for small displacements of up to 0.2, the optical spectra are virtually insensitive to the specific value of Δ_{2p}. The last term in equation (3) accounts for the dipole coupling between 1s and 2p excitons through the phonon mode with a coupling strength $\hbar\Omega$.

The dipole coupling between excitons and phonons, $-\vec{\mu}_{1s,2p}\cdot\vec{E}_{ph}$ is determined by the transition dipole moment $\vec{\mu}_{1s,2p} = \left\langle 2p\right|\vec{\mu}\left|1s\right\rangle$ between the two exciton states and by the electric field associated with the optical phonon mode. The exciton-phonon coupling in equation (3) is introduced as the coupling of excitons to a quantized phonon field[13]. In analogy to light modes, the quantized phonon field[13] mode at a point $\vec{r}$ in the crystal can be expressed as $\vec{E}_{ph}(\vec{r}) = iE_{ph_0}\left[\vec{f}(\vec{r})b - \vec{f}^*(\vec{r})b^\dagger\right]$, with $E_{ph_0} = \sqrt{\dfrac{\hbar\omega_v}{2\varepsilon_0\varepsilon V}}$ being the vacuum field amplitude of the phonon field, ε_0 is the vacuum permittivity and ε the dielectric function of the crystal. The volume of the system is V and $\vec{f}(\vec{r})$ is a dimensionless complex vector field that accounts for

the local amplitude and polarization of the field mode[13,14]. The coupling strength can then be expressed in terms of the vacuum Rabi energy $\hbar\Omega = -i\sqrt{\frac{\hbar\omega_\nu}{2\varepsilon_0\varepsilon V}}\left(\vec{\mu}_{1s,2p}\cdot\vec{f}\right)$.

2.2.1 Estimation of the coupling strength. In the simulations we take $\hbar\Omega \approx \mu_{1s,2p}E_{ph}$. To estimate the amplitude of the transition dipole moment $\mu_{1s,2p}$, we calculate the overlap integral between hydrogen-type 1s and 2p exciton wavefunctions with an exciton radius of $a_X = 3.5\,\text{nm}$ for $CsPbBr_3$ according to the literature[12]. This results in $\mu_{1s,2p} = 3\cdot 10^{-28}\,Cm \approx 90D$. An estimate of the phonon field amplitude is obtained by considering the polarization field amplitude $E_{pol} = P/\varepsilon_0(\varepsilon - 1)$ induced by the Pb-Br_3 dipoles oscillating at the phonon frequency of 50 cm^{-1}. Here $P = qd/V$ is the polarization density, d the displacement in units of length, and V the unit cell volume. The lattice parameter $a = 0.8\,\text{nm}$[15] and the relative dielectric constant $\varepsilon = 7.3$[16] are taken from the literature. This gives a phonon field amplitude of $E_{ph} \simeq 4.4\cdot 10^6$ V/m. With this, we estimate a coupling strength of $\hbar\Omega = \mu_{1s,2p}E_{ph} \approx 8\,\text{meV}$. This coupling strength is ~27% of the 1s-2p electronic splitting $\Delta E_{1s,2p}$, pointing to an ultrastrong coupling regime between the intra-exciton (1s-2p) excitation and the phonon mode. The numerical 2DES simulations shown in Fig. 4 of the manuscript give reasonable agreement with the experiments when taking a coupling strength of $\hbar\Omega = 3.7\,\text{meV}$, which is $\hbar\Omega \sim 0.12\Delta E_{1s,2p}$, at the onset of ultrastrong coupling.

2.2.2 Details of the 1s-2p-phonon coupling term and Rabi oscillations. The last term in equation (3) is fundamental to explain our experimental results. It consists of two distinct coupling pathways between 1s and 2p excitons that arise from co- and counter-rotating terms in the interaction Hamiltonian in equation (3). To underline the two components, we expand the interaction term as

$$\begin{aligned}&\hbar\Omega\left(b^\dagger + b\right)\left(c_{1s}^\dagger c_{2p} + c_{2p}^\dagger c_{1s}\right) = \\ &= \hbar\Omega\left(bc_{2p}^\dagger c_{1s} + b^\dagger c_{1s}^\dagger c_{2p}\right) + \hbar\Omega\left(b^\dagger c_{2p}^\dagger c_{1s} + bc_{1s}^\dagger c_{2p}\right) = H_{c,RWA} + H_{c,CR}\end{aligned} \quad . \qquad (4)$$

The co-rotating terms $H_{c,RWA} = \sum_\nu \hbar\Omega_\nu\left(|2p,\nu\rangle\langle 1s,\nu+1| + |1s,\nu+1\rangle\langle 2p,\nu|\right)$, with $\hbar\Omega_\nu = \hbar\Omega\sqrt{\nu+1}$, induce transitions between $|1s,\nu+1\rangle$ and $|2p,\nu\rangle$ by phonon absorption or

stimulated emission. These terms conserve the total number of excitations in the system[17] and survive the rotating wave approximation (RWA) that is applied in the Jaynes-Cumming model for light-driven systems[13]. Thus we label them "RWA couplings" in the following. The dynamics induced by such RWA couplings usually dominate when the bare exciton and the field mode are nearly resonant[17]. In our case instead, the bare electronic 1s-2p energy splitting $\Delta E_{1s,2p}$ is ~5 times larger than the phonon energy. Thus, the phonon field drives the excitonic 1s-2p transition off-resonantly. As such, the counter-rotating (CR) terms of the interaction in equation (4), $H_{c,CR} = \sum_{\nu} \hbar\Omega_{\nu} \left(|2p,\nu+1\rangle\langle 1s,\nu| + |1s,\nu\rangle\langle 2p,\nu+1| \right)$, can no longer be neglected and the RWA is no longer justified. These CR terms describe transitions between $|1s,\nu\rangle$ and $|2p,\nu+1\rangle$ states that do not conserve the total number of excitations[17,18].

In the ultrastrong matter-field coupling regime, i.e., for $\hbar\Omega \geq 0.1\Delta E_{1s,2p}$, both co- and counter-rotating terms in the interaction Hamiltonian are relevant for the system dynamics[18]. They induce coherent oscillations between the 1s and 2p populations by a sequence of excitation-number-conserving and non-conserving absorption and stimulated emission processes. These off-resonant phonon-induced intra-exciton Rabi oscillations give rise to the fast, 107-fs-period oscillations seen in Fig. 3a and to the subpeak structure around the exciton peak in the 2DES maps shown in Figs. 1 and 2 of the manuscript.

2.2.3 Rabi oscillation periods induced by the RWA terms. To estimate the oscillation periods of those Rabi oscillations, we first consider only the RWA terms of the interaction between 1s and 2p in equation (4). In this case, analogous to the Jaynes-Cummings model[13], the lowest-lying $|1s,0\rangle$ state does not participate in the coupling and thus the first eigenstate coincides with the uncoupled state $|1s,0\rangle$ with energy $\hbar\omega_{1s}$, whereas the other eigenstates arise from pairwise mixing of $|1s,\nu+1\rangle$ and $|2p,\nu\rangle$. As such, except for the lowest-lying state, the RWA couplings lead to pairs of 1s-2p-exciton-phonon hybrid states with energies $E^{\pm}_{RWA} = \frac{E_{1s,\nu+1} + E_{2p,\nu}}{2} \pm \frac{1}{2}\sqrt{4\left(\hbar\Omega_{\nu}\right)^2 + \Delta E^2_{RWA}}$, with the energetic detuning $\Delta E_{RWA} = \Delta E_{1s,2p} - \hbar\omega_{\nu} = 23.7$ meV. Because of the weak displacement Δ_{1s} of the 1s exciton potential energy surface, the resonant optical excitation, inducing transitions from the system ground to the excited state, mainly populates $|1s,0\rangle$ and only weakly $|1s,1\rangle$, whereas the population of the higher-lying states $|1s,\nu>1\rangle$ is negligible (Fig. S7b). Due to the RWA

coupling terms, upon optical excitation of the 1s exciton, oscillations between the populations of 1s and 2p manifolds take place with a transfer probability from 1s to 2p given by[19] $P_{RWA,\nu}(t)=\frac{4(\hbar\Omega_\nu)^2}{4(\hbar\Omega_\nu)^2+\Delta E_{RWA}^2}\sin^2\left(\frac{\sqrt{4(\hbar\Omega_\nu)^2+\Delta E_{RWA}^2}}{2\hbar}t\right)$ as a function of time t. For each ν, the periods of these oscillations $T_{\nu_{RWA}}=\frac{2\pi\hbar}{\sqrt{4(\hbar\Omega_\nu)^2+\Delta E_{RWA}^2}}$ depend on the energetic splittings $\sqrt{4(\hbar\Omega_\nu)^2+\Delta E_{RWA}^2}$ between the eigenstates pairs. These splittings, however, result in slower oscillation periods than the 107-fs ones observed in our experiments. In fact, for $\hbar\Omega=3.7$ meV and $\nu=0$ describing the coupling of $|1s,1\rangle$ and $|2p,0\rangle$, we get an energy splitting of 24.8 meV (167 fs). For higher-lying vibronic states with quantum number $\nu>0$, the energy splitting increases only slightly, e.g., we get 27 meV (153 fs), for the coupling of $|1s,3\rangle$ and $|2p,2\rangle$. Moreover, since the RWA terms cannot induce couplings between the $|1s,0\rangle$ state and the 2p manifold, no oscillations are expected for the population of the lowest-lying state. This is confirmed by simulating the population dynamics of the vibronic excited states $n_{i,\nu}$, with i =1s, 2p, and $\nu=0,1,2,3,...$, taking only the RWA terms of the interaction into account (Fig. S10). As such, the RWA terms cannot explain the experimentally observed 310 cm^{-1} oscillations in Figs. 1-3 of the manuscript.

2.2.4 Rabi oscillation periods induced by the full interaction. In the full 1s-2p-phonon interaction (equation 4), additional coupling pathways become allowed due to the CR terms. This leads to the hybridization of multiple 1s and 2p vibronic states, and not only of pairs as in the RWA case (see section 2.2.3). Importantly, now also $|1s,0\rangle$ becomes involved in the coupling through the CR terms. To estimate the oscillation periods, we perform a numerical diagonalization of the 1s-2p Hamiltonian. We have checked that, to this end, it is sufficient to consider the first five vibrational levels for each electronic state, since Δ_{1s} is small. The absolute values of the amplitude of the eigenvector components for the first two eigenstates are depicted in Fig. S11a-b as a function of the coupling strength. For very small coupling strengths, the lowest-lying eigenstate (Fig. S11a) results from a mixture of mainly $|1s,0\rangle$ and $|2p,1\rangle$, with contributions of other higher-lying 1s and 2p vibronic states being negligible. The role of these higher-lying states, however, becomes more and more important as the coupling strength increases. For the lowest-lying eigenstate (Fig. S11a), for example, the amplitude of the

eigenvector components shows that, while $|1s,0\rangle$ and $|2p,1\rangle$ depend linearly on the coupling strength over a broad range up to about 5 meV, a quadratic and cubic dependence is seen for the contribution of the $|1s,2\rangle$ and the $|2p,4\rangle$ states, respectively. This means that, in first-order perturbation, the most relevant contribution to the lowest-lying eigenstate arises from the coupling between $|1s,0\rangle$ and $|2p,1\rangle$ due to a CR term, whereas higher-lying $|1s,2\rangle$ and $|2p,4\rangle$ states become relevant only at higher perturbation orders. Similar arguments apply for the contributions of the CR coupling between $|1s,1\rangle$ and $|2p,2\rangle$ and the RWA coupling between $|1s,1\rangle$ and $|2p,0\rangle$ to the second eigenstate (Fig. S11b). As such, in the limit of vanishingly small coupling strengths, we can use a simplified perturbative picture to rationalize the main contributions to the splittings, and thus to the population oscillations, by considering the sequential absorption and stimulated emission processes induced by the RWA and CR terms. Fig. S11c schematically shows the first two of these subsequent absorption (solid lines) and stimulated emisssion (dashed lines) processes with the CR-induced transitions highlighted in blue and the RWA ones in red. We further highlight with dark colors the three relevant first order contributions to the population dynamics, whereas higher order perturbation contributions are in lighter colors. In this approximation, and since $|1s,0\rangle$ is the optically most populated state, we recognize again that the relevant contribution to the splittings reflects the CR coupling between $|1s,0\rangle$ and $|2p,1\rangle$. The results in Fig. S11a also suggest that, for the herein estimated coupling strength of 3.7 meV (dashed line), it is reasonable to assume that the relevant energy splitting reflects mainly the CR coupling pathway between $|1s,0\rangle$ and $|2p,1\rangle$. This approximation also applies to the coupling between $|1s,1\rangle$ and $|2p,2\rangle$ states. As such, we can estimate the periods of the population oscillations due to the CR terms by an approximation of the energy splittings arising from a linear perturbation to the bare energy detuning ΔE_{CR} between the uncoupled $|1s,\nu\rangle$ and $|2p,\nu+1\rangle$ states. This bare energy detuning is $\Delta E_{CR} = \Delta E_{1s,2p} + \hbar\omega_\nu = 36.3\,\text{meV}$. Since for $\hbar\Omega = 3.7\,\text{meV}$ we can neglect, at least in first-order perturbation, the contribution of the higher-lying states to the coupling, we approximate the energy splitting between $|1s,\nu\rangle$ and $|2p,\nu+1\rangle$ upon coupling through the CR terms by expanding the energy splitting of the eigenstates of a coupled two-level-system in a Taylor series and truncating it to the first order, i.e,. $\sqrt{4\left(\hbar\Omega_\nu\right)^2 + \Delta E_{CR}^2} \approx \Delta E_{CR} + \frac{4\left(\hbar\Omega_\nu\right)^2}{2\Delta E_{CR}}$. This way,

we obtain an estimate for the energy splitting of 37.1 meV (111 fs) for the coupling between $|1s,0\rangle$ and $|2p,1\rangle$, which nicely matches the experimentally observed ~107-fs oscillations.

Finally, we compare these considerations to a full numerical simulation of the population dynamics of the excited vibronic states $n_{i,v}$ (Fig. S12) under the 1s-2p-phonon interaction Hamiltonian in equation (4). This result shows that oscillations in the populations of $|1s,0\rangle$ and $|2p,1\rangle$ with a period of ~109 fs (307 cm^{-1}) dominate the dynamics.

2.3 Numerical simulation of the 2DES maps. The numerical simulations of the 2DES maps are performed using a nonperturbative approach to the solution of the master equation for the density matrix ρ as we have previously reported in Refs[20,21]. Briefly, we numerically solve the master equation in the Lindblad form[22]

$$\dot{\rho} = -\frac{i}{\hbar}[H,\rho] + \sum_m \mathcal{L}_m(\rho) \tag{5}$$

with the total Hamiltonian $H = H_0 + H_I(t)$ describing the free evolution of the system H_0, as defined in sections 2.1 and 2.2, for the DHO model and for the extended DHO with 1s-2p-phonon coupling, respectively, and its light-matter interaction $H_I(t) = -\hat{\mu}\sum_{j=1}^{3} E_j(t)$ in semiclassical point dipole approximation. Here $\hat{\mu}$ is the dipole operator and each optical field $E_j(t)$ represents one of the three external laser pulses in the 2DES excitation sequence. Each $E_j(t) = A_j e^{-2\ln 2\left(\frac{t-t_{0,j}}{t_w}\right)^2} \cos\left[\omega\left(t-t_{0,j}\right)+\varphi_j\right]$ is taken as a Gaussian pulse, with amplitude A_j, duration $t_w = 5$ fs defined as the full-width-at-half-maximum of the field intensity profile, time delay $t_{0,j}$ of the *j*-th pulse with respect to the third (probe) pulse which is centered at $t_{0,3} = 0$ fs, and phase shift φ_j used for phase-cycling[20]. In equation (5), we model phenomenologically the dissipation arising from the interaction of the system with the environment adopting the Lindblad formalism with the superoperators[22] $\mathcal{L}_m(\rho) = \gamma_m\left(V_m\rho V_m^\dagger - \frac{1}{2}\left\{V_m^\dagger V_m,\rho\right\}\right)$, with γ_m describing the damping rate for the *m*-th damping process. Since population and vibrational relaxation take place on long timescales of >50 ps and ~8 ps, respectively, they do not influence the dynamics on the timescale of the simulations presented here and in the main manuscript. Therefore, in the simulations we only consider electronic dephasing. To this aim, we define

$\gamma = 1/T_2$ as the electronic dephasing rate, with $T_2 = 200$ fs being the electronic dephasing time estimated from the width of the exciton diagonal peak in our experimental data, and $V_d^\dagger = V_d = |1s\rangle\langle 1s| + |2p\rangle\langle 2p|$ the creation and annihilation operators for electronic dephasing. For these calculations, we choose to work in the basis of displaced harmonic oscillators. Therefore, we perform a basis transformation using the unitary displacement operator $D(\Delta_i) = \exp\left[\Delta_i\left(b^\dagger - b\right)\right]$, with Δ_i being the dimensionless displacement of the *i*-th excited state potential energy surface along the vibrational coordinate, as defined in sections 2.1 and 2.2. Since only the 1s potential has a finite displacement with respect to the ground state, in the displaced basis, the vibrational wavefunctions associated with the 1s state become DHO wavefunctions centered around Δ_{1s}.

To calculate the 2DES maps, we numerically solve equation (5) using a nonperturbative approach[20,21] to obtain $\rho(\tau, T, t)$ as a function of τ, T, and of the simulation time *t*. From this, we calculate the total polarization $P(\tau, T, t) \approx Tr\left\{\hat{\mu}\rho(\tau, T, t)\right\}$. We then apply a phase cycling algorithm[21] to obtain the directional dependence of the nonlinear part of the polarization under the phase-matched condition mimicking the experiment, and finally subtract the linear contribution due to the interaction with the probe pulse to yield the third-order nonlinear polarization $P^{(3)}(\tau, T, t)$. To obtain the 2DES maps in reflection, we perform a Fourier transform of the calculated polarization along the simulation time t for each τ and T, and we then calculate the nonlinear optical susceptibility $\chi(\tau, T, \omega) \approx \dfrac{P^{(3)}(\tau, T, \omega)}{E_0(\omega)}$. The reflection spectrum is approximated using the Fresnel equations for the reflection coefficient with the refractive index $n(\omega) = \sqrt{\varepsilon(\omega)} \approx \sqrt{\varepsilon_{BG} + \chi(\omega)}$, where ε_{BG} is the constant non-resonant background contribution to the total dielectric function. The differential spectrum is computed as for the experimental data (see Methods). Hence, at each waiting time T, a Fourier transform of the differential spectra along τ results in energy-energy maps for excitation E_X and detection E_D energy.

Supplementary Figures

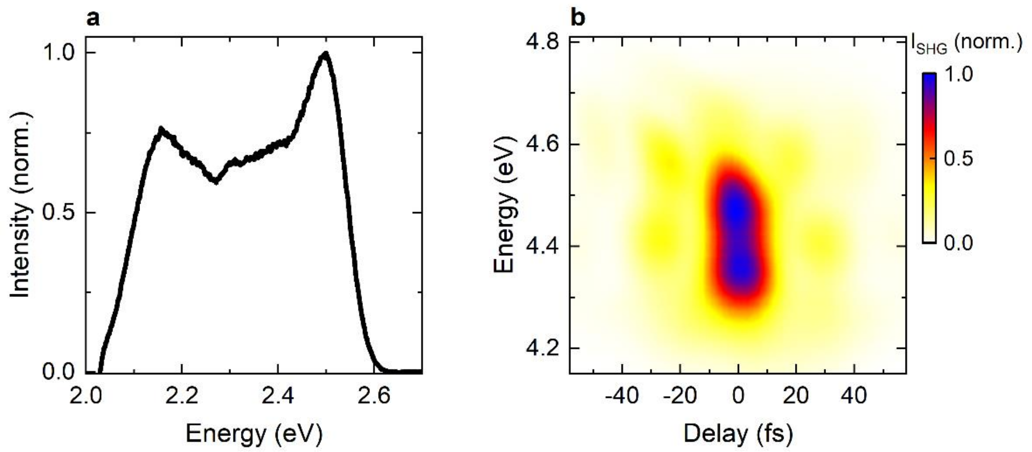

Fig S1. a, NOPA spectrum used to excite and probe the $CsPbBr_3$ crystals. The optical pulses cover the near band-edge absorption of excitons and free carriers with an excess excitation energy of ~ 200 meV above the bandgap. **b**, SHG-FROG map of the cross-correlation between the pump and probe beams at the sample position provides a time resolution of ~13 fs.

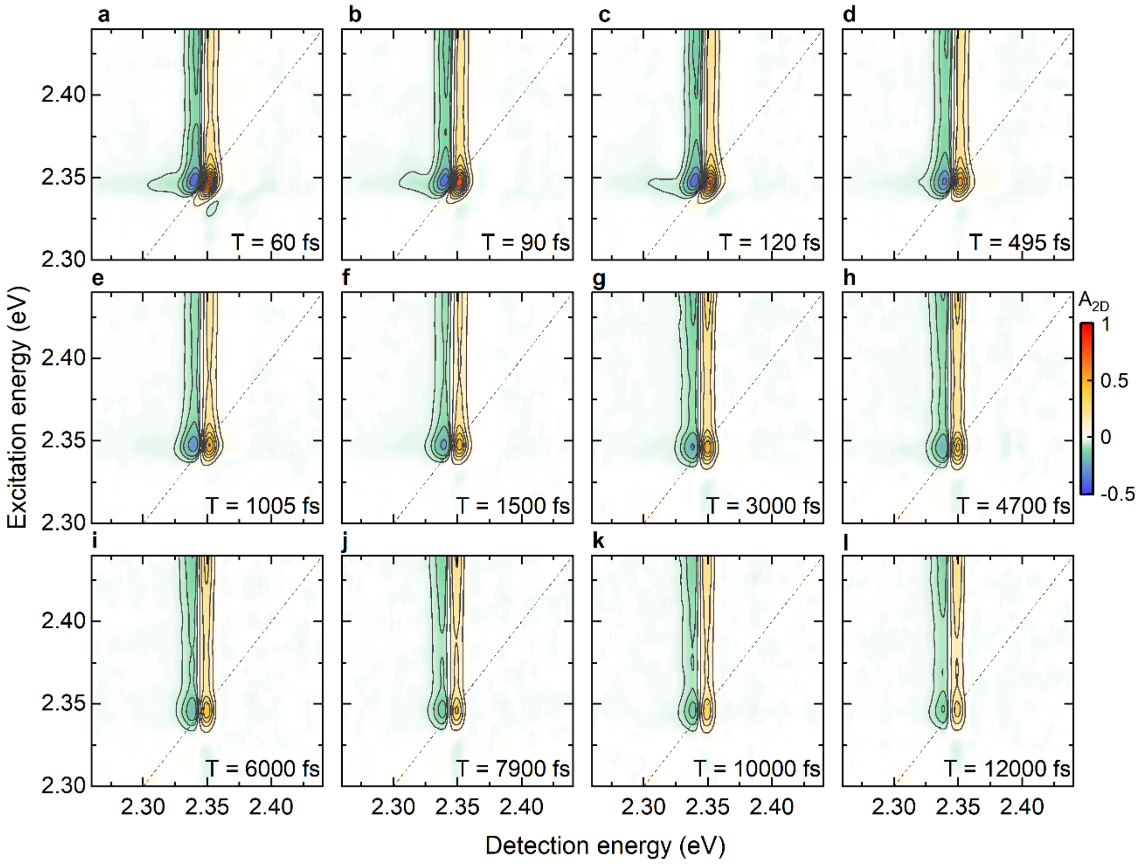

Fig S2. a-l, 2DES maps of $CsPbBr_3$ single crystals at 20 K for selected waiting times T between 60 fs and 12 ps. The vertically elongated free-carrier-induced cross-peak for excitation >2.40 eV in the free-carrier continuum shows a slow amplitude decay (see also Fig. S3), but no time-dependent lineshape changes or spectral peak shifts, as one would expect if carrier relaxation was the dominant nonlinearity[20]. This indicates that, at 20 K under our excitation conditions, relaxation within the free-carrier continuum is not the dominant contribution to the elongated cross-peak.

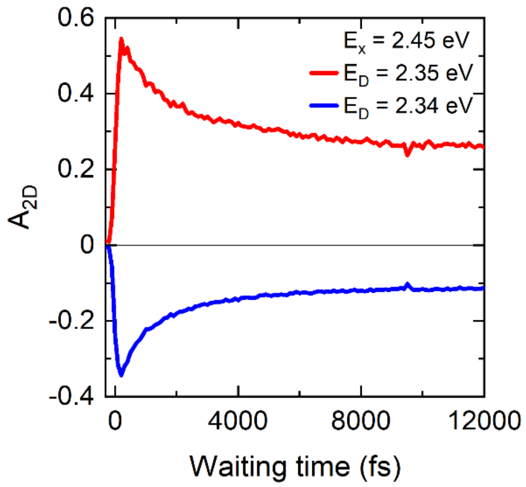

Fig S3. Waiting time dynamics of the vertically elongated dispersive cross-peak in $CsPbBr_3$ single crystals at 20 K for excitation at E_X = 2.45 eV into the free-carrier continuum and detection E_D = 2.35 eV (red) and E_D = 2.34 eV (blue) on the high and low energy side of the main exciton resonance. In contrast to the exciton peaks, the dynamics of this elongated cross-peak show only a slow bi-exponential decay on timescales of ~2.5 ps and >50 ps, but no oscillations. The fast decay is mainly given by diffusion out of the excitation volume[20].

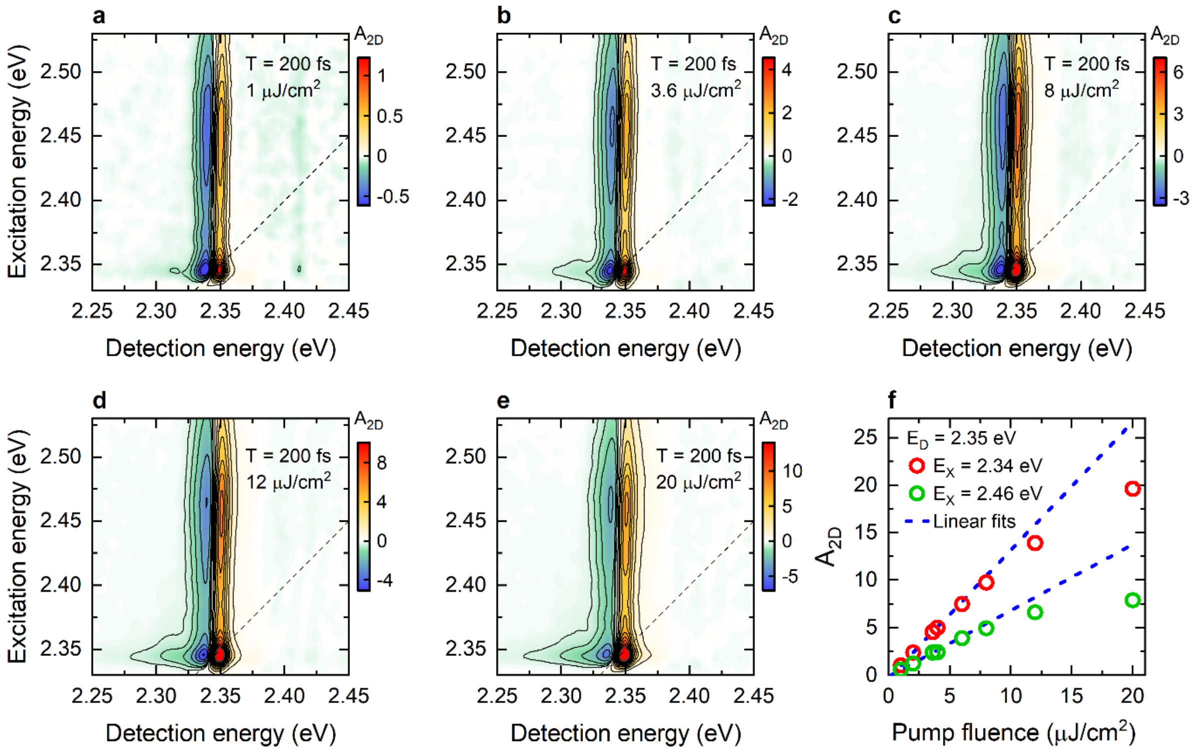

Fig S4. a-e, 2DES maps of $CsPbBr_3$ single crystals at 20 K and at a fixed waiting time T = 200 fs for increasing pump fluences between 1μJ/cm^2 and 20 μJ/cm^2. Within this fluence range, the shape of the 2DES map remains essentially unchanged. With increasing fluence, we observe a broadening of the peak lineshape along the detection energy E_D suggesting excitation-induced dephasing. **f**, 2DES signal amplitude A_{2D} at excitation energies of E_X = 2.34 eV (red) and E_X = 2.46 eV (green), and E_D = 2.35 eV as a function of the pump fluence show a linear correlation for fluences <10 μJ/cm^2 for both the exciton diagonal peak (red) and the free-carrier-induced cross-peak (green). The dashed blue lines are linear fits to the data. The 2DES data shown in the main manuscript are recorded with a pump fluence of 4 μJ/cm^2.

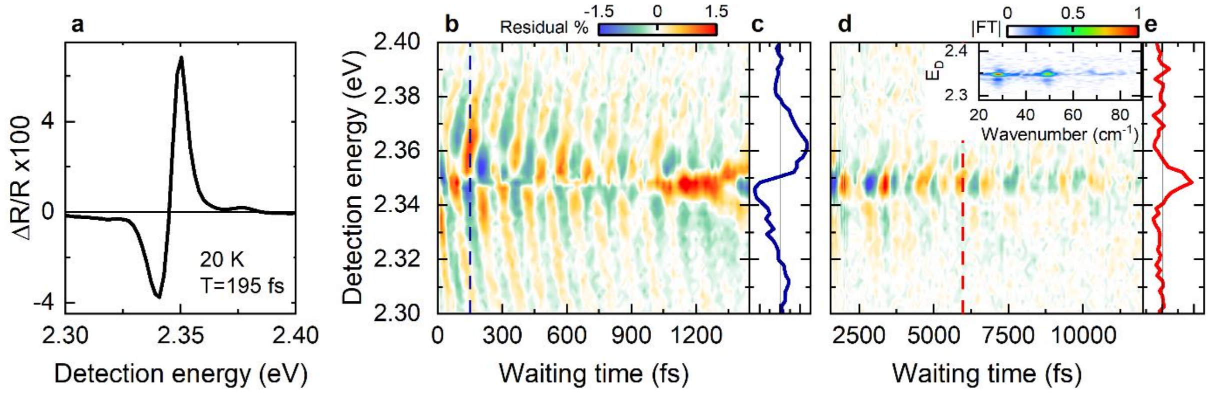

Fig S5. a, Differential reflectivity $\Delta R/R$ spectrum of $CsPbBr_3$ at 20 K and at a waiting time T = 195 fs for resonant excitation of the exciton. **b-e**, Residual maps showing the oscillatory amplitude modulation of the $\Delta R/R$ after subtraction of the slowly decaying multi-exponential background. **b-c**, For T< 2 ps (b) the residuals show fast, 107 fs (310 cm^{-1}) oscillations with (c) a dispersive spectral profile along the detection energy indicating Rabi oscillations. **d-e,** For T > 2 ps (d), slower oscillations at ~30 cm^{-1} and 50 cm^{-1} (FT in the inset) with (e) an absorptive spectral profile are observed matching instead the expectations of a displaced harmonic oscillator model and thus that those low-frequency oscillations arise from coherent phonon wavepacket motion.

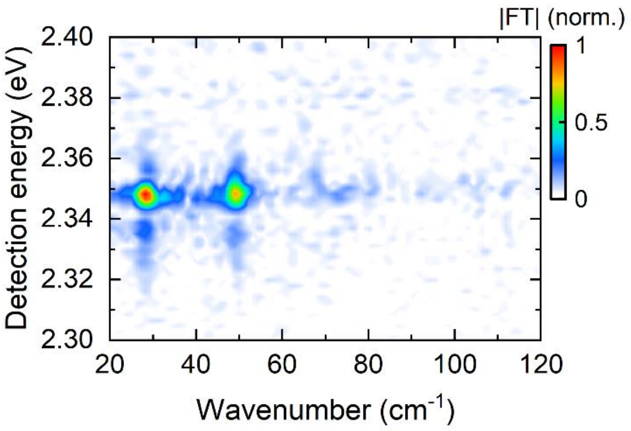

Fig S6. Normalized Fourier transform amplitude of the residual map in Fig. S5d on the entire detection energy range shows two low frequency components at 28 cm^{-1} and 50 cm^{-1}.

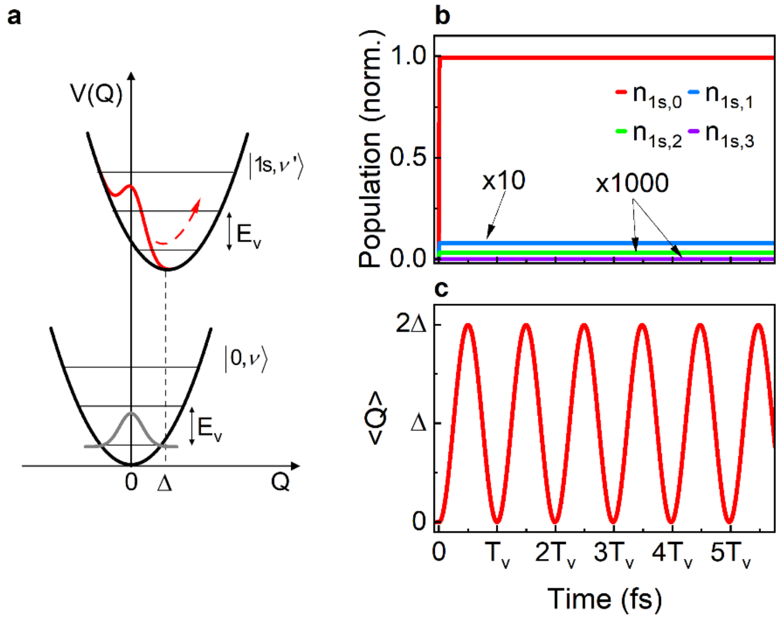

Fig S7. Displaced harmonic oscillator model. **a**, Scheme showing the ground $|0,\nu\rangle$ and excited exciton bright $|1s,\nu'\rangle$ states with ν and ν' the vibrational quantum number of ground and excited states, respectively. The ground and excited state are assumed as harmonic potential energy surfaces as a function of the dimensionless phonon coordinate Q , with E_V the phonon mode energy. The coupling of the 1s exciton to the phonon mode induces a displacement Δ of the 1s potential energy surface along Q. **b**, For a small displacement of 0.1, the impulsive resonant optical excitation populates mainly the $|1s,0\rangle$ state, whereas the population of the higher-lying vibronic states $|1s,\nu'>0\rangle$ is negligible. **c**, The impulsive excitation launches coherent phonon wavepacket motion on the 1s potential energy surface. For sufficiently short excitation pulses, i.e., with a pulse duration that is much shorter than the oscillation period of the phonon mode $T_V = 2\pi\hbar/E_V$, the expectation value of the phonon coordinate $\langle Q\rangle$, describing the motion of the center of mass of the phonon wavepacket, shows a cosine-like function with period T_V .

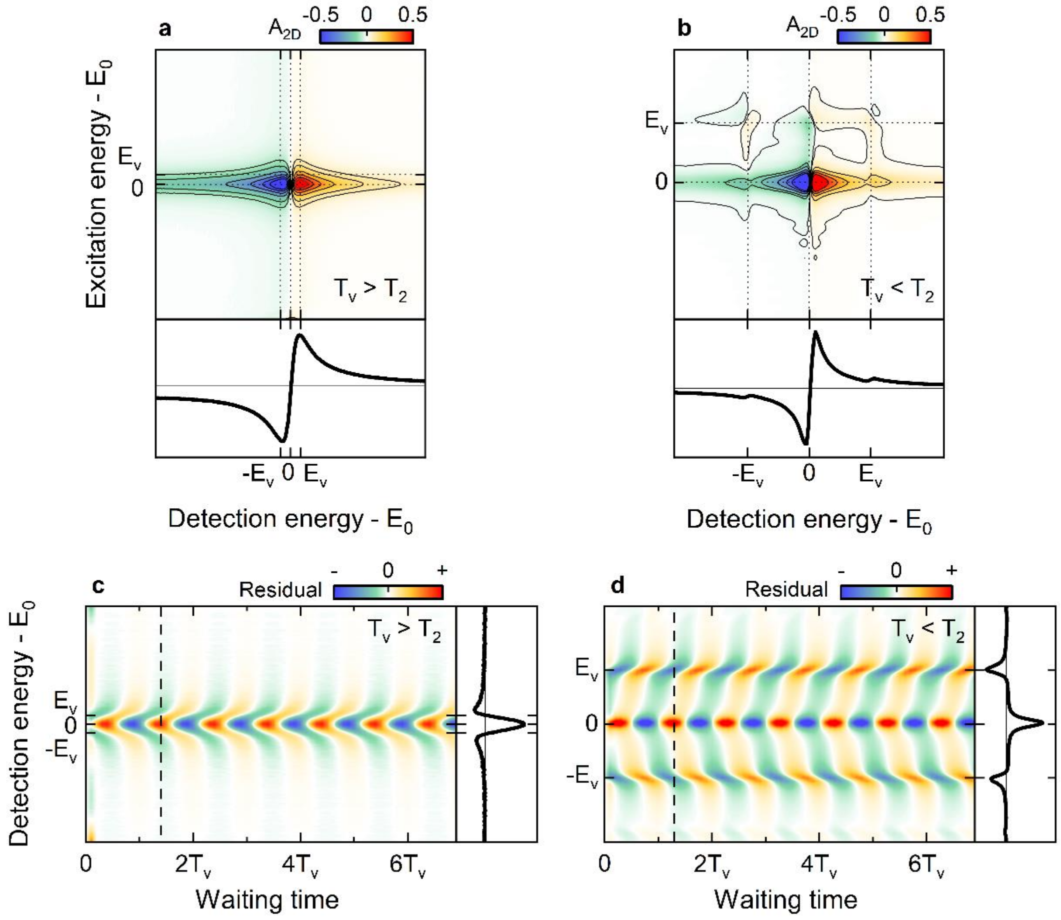

Fig S8. Signatures of phonon wavepacket motion in 2DES in reflection based on the displaced harmonic oscillator model. **a-b**, Simulated 2DES maps at a selected waiting time T and cross-cuts at a fixed excitation energy corresponding to the exciton main peak E_0. Both excitation E_X and detection E_D axes are shifted by E_0. Dotted lines in (a-b) indicate the grid-like structure which is induced in the 2DES map by the excitation of phonon wavepackets[21]. The spacings between the peaks along E_X and E_D are defined by the phonon energy E_V. Phonon side-peaks along E_D arise from bleaching (side-peak at E_V) and stimulated emission (side peak at $-E_V$) pathways between ground and excited state. Depending on the vibrational oscillation period T_V of the mode being (a) larger or (b) shorter than the exciton electronic dephasing time T_2, the phonon side-peaks can be (a) hidden within the homogeneous lineshape limited by T_2 or (b) they can be clearly distinguished from the main resonance, as further highlighted by taking cross-sections at $E_X = 0$ eV. **c-d**, Residual maps as a function of T obtained as in the experiment show an absorptive-like spectral profile along E_D with the maximum amplitude of the oscillation in the zero-crossing of the 2DES cross-section along E_D (cf. panels a-b) for both (c) $T_V > T_2$ and (d) $T_V < T_2$.

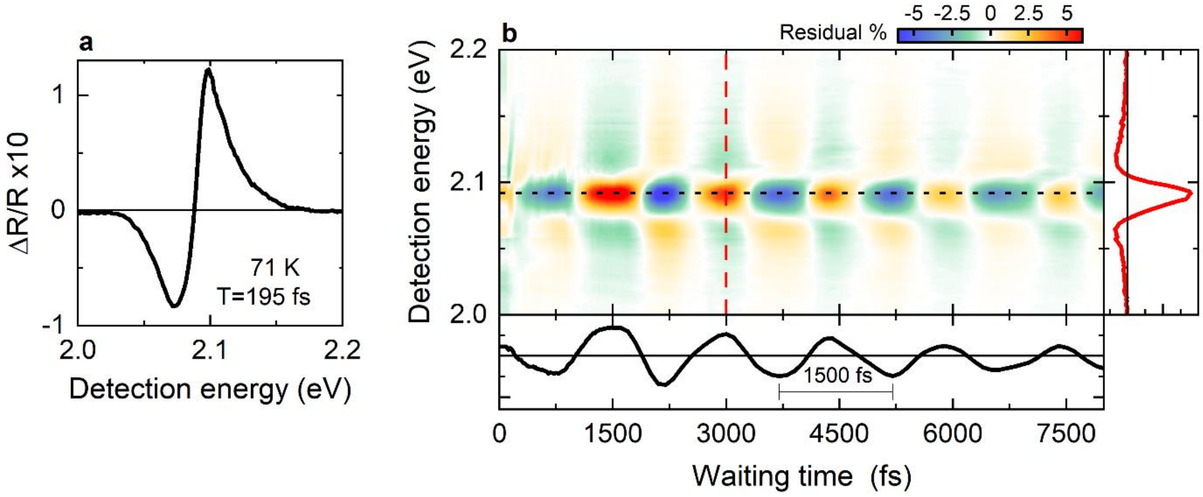

Fig S9. Differential reflectivity $\Delta R/R$ of $(BA)_2(MA)_{n-1}Pb_nI_{3n+1}$, n = 3, two-dimensional crystals at 71 K. **a**, The differential reflectivity spectrum at T = 195 fs shows a dispersive lineshape with a zero-crossing at ~2.09 eV corresponding to the 1s exciton resonance[23]. **b**, The residual map reveals persistent oscillations with a dominant period of 1500 fs (~ 22 cm^{-1}) up to at least 8 ps. The map also reveals a clear absorptive spectral profile along the detection energy axis (inset on the right shows a cross-section). This is in excellent agreement with predictions of a displaced harmonic oscillator model and thus indicates that the oscillations arise from coherent phonons. The oscillation frequency matches low frequency phonon motions of the Pb-I lattice[24]. The bottom inset shows a cross-cut at the detection energy indicated by the horizontal dotted black line in the residual map.

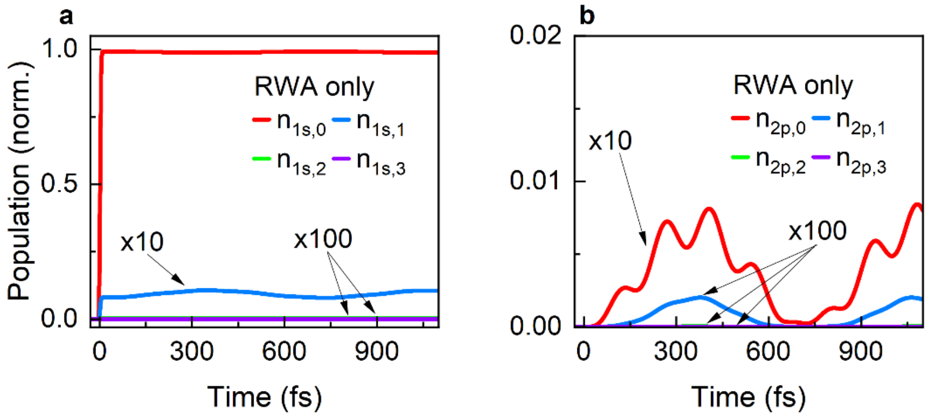

Fig. S10. a-b, Simulated dynamics of the (a) 1s and (b) 2p vibronic populations $n_{i,\nu}$, with i = 1s, 2p, and $\nu = 0,1,2,3$, within the RWA. Since the RWA terms cannot induce couplings between $|1s,0\rangle$ and the 2p manifold, $n_{1s,0}$ shows no oscillations (a, red line). For the higher-lying 1s vibronic states and (b) the entire 2p manifold, the populations feature negligible amplitude oscillations with periods of >150 fs, slower than the 107 fs oscillations observed in our experiment.

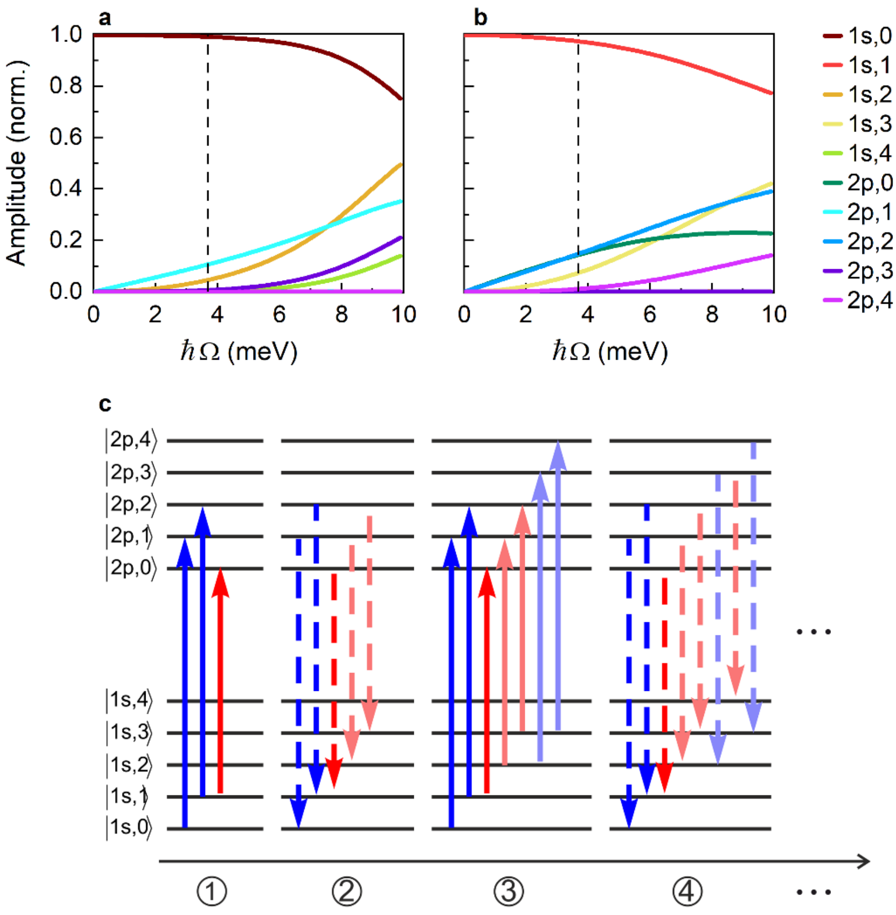

Fig. S11. a-b, Absolute values of the amplitude components of (a) the first and (b) the second lowest-lying eigenvector obtained by numerical diagonalization of the full interaction 1s-2p-Hamiltonian as a function of the coupling strength $\hbar\Omega$. **c**, Schematic illustration of the first two subsequent absorption (1 and 3, solid arrows) and stimulated emission processes (2 and 4, dashed arrows) following the optical excitation of the exciton 1s in the limit of vanishingly small coupling strength. For clarity of visualization, the ground state is not shown and the energetic detuning ($\Delta E_{1s,2p} = 30$ meV) between the 1s and 2p manifolds is not to scale. Blue and red arrows indicate CR-induced and RWA-induced pathways, respectively. Due to the small displacement of the 1s exciton potential energy surface, the optical excitation mainly populates $|1s,0\rangle$ and only weakly $|1s,1\rangle$. As a consequence, the transitions from and to higher-lying vibronic states $|1s,v>1\rangle$ to 2p, which take place after multiple interactions (3, 4, …), will not significantly contribute to the population dynamics. Therefore, we have highlighted with dark colors only the lowest CR-induced (dark blue) and RWA-induced (dark red) pathways which are most important for the population oscillations.

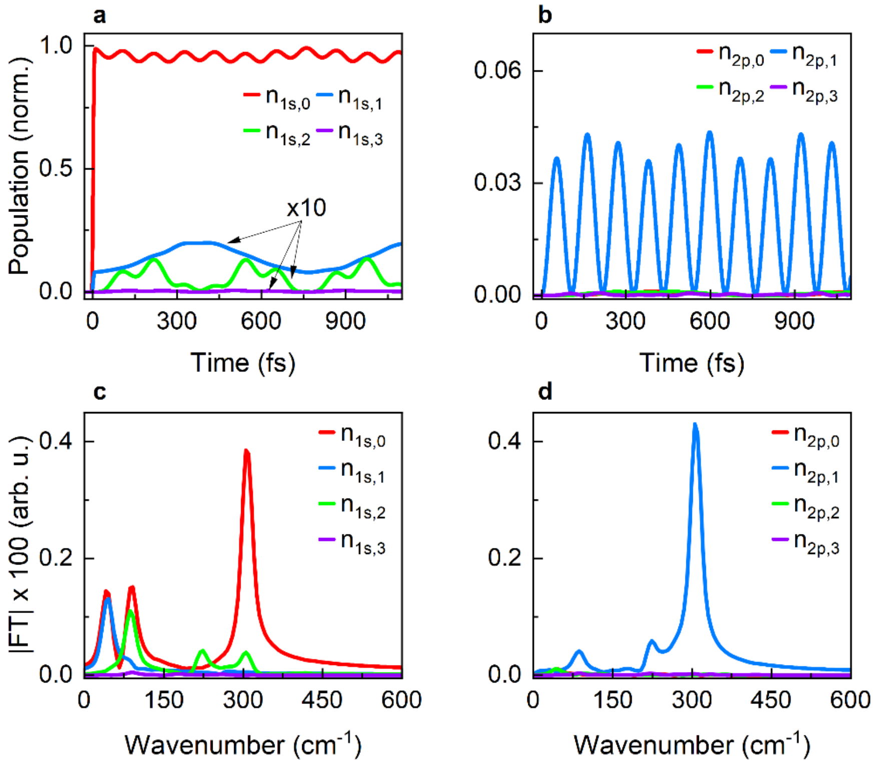

Fig. S12. a-b, Simulated dynamics of the (a) 1s and (b) 2p vibronic populations $n_{i,\nu}$, with i = 1s, 2p, and $\nu = 0,1,2,3$, based on the full interaction 1s-2p-Hamiltonian. Fast oscillations of $n_{1s,0}$ (a, red line) and $n_{2p,1}$ (b, blue line) with a period of ~109 fs dominate the population dynamics of the 1s and 2p manifolds, respectively. These population oscillations are induced by CR terms of the interaction between $|1s,0\rangle$ and $|2p,1\rangle$. Slower oscillatory features in the population of higher-lying vibronic states have negligible amplitude. **c-d**, Fourier transforms of the oscillatory part of the corresponding $n_{i,\nu}$ in (a) and (b).

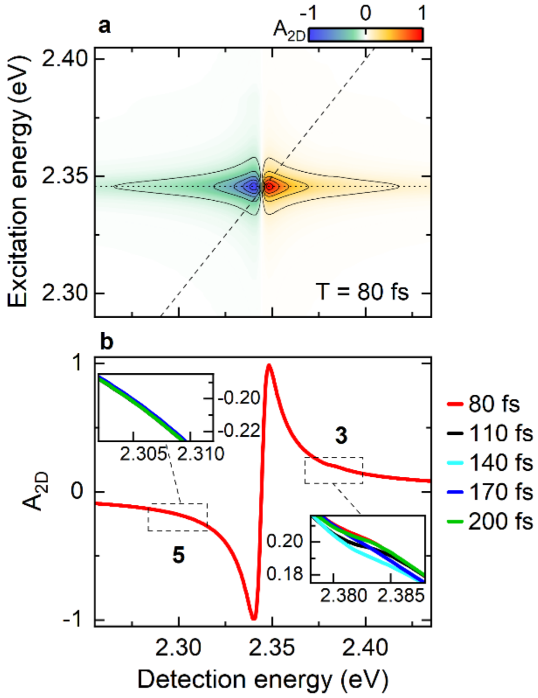

Fig. S13. a, Exemplary simulated 2DES map (linear scale plot) at a waiting time T = 80 fs showing the dispersive exciton peak at 2.345 eV. **b**, Cross-sections at a fixed excitation energy at 2.345 eV (dotted horizontal line in a), for selected waiting times between 80 and 200 fs, reveal a weak oscillating side-peak at a detection energy of ~2.38 eV (3, inset), on the high-energy side of the exciton main resonance, but no side-peaks on the low-energy side (5, inset), in agreement with the experimental data.

Supplementary References

1 Zhou, X. & Zhang, Z. Electron–phonon coupling in CsPbBr3. *AIP Advances* **10**, 125015 (2020).

2 Guo, P. *et al.* Polar Fluctuations in Metal Halide Perovskites Uncovered by Acoustic Phonon Anomalies. *ACS Energy Letters* **2**, 2463-2469 (2017).

3 Kresse, G. & Furthmüller, J. Efficiency of ab-initio total energy calculations for metals and semiconductors using a plane-wave basis set. *Computational Materials Science* **6**, 15-50 (1996).

4 Kresse, G. & Furthmüller, J. Efficient iterative schemes for ab initio total-energy calculations using a plane-wave basis set. *Physical Review B* **54**, 11169-11186 (1996).

5 Kresse, G. & Joubert, D. From ultrasoft pseudopotentials to the projector augmented-wave method. *Physical Review B* **59**, 1758-1775 (1999).

6 Blöchl, P. E. Projector augmented-wave method. *Physical Review B* **50**, 17953-17979, (1994).

7 Baroni, S., de Gironcoli, S., Dal Corso, A. & Giannozzi, P. Phonons and related crystal properties from density-functional perturbation theory. *Reviews of Modern Physics* **73**, 515-562 (2001).

8 Zhang, X. B., Taliercio, T., Kolliakos, S. & Lefebvre, P. Influence of electron-phonon interaction on the optical properties of III nitride semiconductors. *Journal of Physics: Condensed Matter* **13**, 7053-7074 (2001).

9 Franchini, C., Reticcioli, M., Setvin, M. & Diebold, U. Polarons in materials. *Nature Reviews Materials* **6**, 560-586 (2021).

10 Ghosh, D., Welch, E., Neukirch, A. J., Zakhidov, A. & Tretiak, S. Polarons in Halide Perovskites: A Perspective. *The Journal of Physical Chemistry Letters* **11**, 3271-3286 (2020).

11 Park, M. *et al.* Excited-state vibrational dynamics toward the polaron in methylammonium lead iodide perovskite. *Nature Communications* **9**, 2525 (2018).

12 Protesescu, L. *et al.* Nanocrystals of Cesium Lead Halide Perovskites (CsPbX3, X = Cl, Br, and I): Novel Optoelectronic Materials Showing Bright Emission with Wide Color Gamut. *Nano Letters* **15**, 3692-3696 (2015).
13 Haroche, S. & Raimond, J. M. *Exploring the Quantum: Atoms, Cavities, and Photons*. (OUP Oxford, 2013).
14 Kira, M. & Koch, S. W. *Semiconductor Quantum Optics*. (Cambridge University Press, 2011).
15 López, C. A. *et al.* Crystal Structure Features of CsPbBr3 Perovskite Prepared by Mechanochemical Synthesis. *ACS Omega* **5**, 5931-5938 (2020).
16 Yang, Z. *et al.* Impact of the Halide Cage on the Electronic Properties of Fully Inorganic Cesium Lead Halide Perovskites. *ACS Energy Letters* **2**, 1621-1627 (2017).
17 Frisk Kockum, A., Miranowicz, A., De Liberato, S., Savasta, S. & Nori, F. Ultrastrong coupling between light and matter. *Nature Reviews Physics* **1**, 19-40 (2019).
18 Forn-Díaz, P., Lamata, L., Rico, E., Kono, J. & Solano, E. Ultrastrong coupling regimes of light-matter interaction. *Reviews of Modern Physics* **91**, 025005 (2019).
19 Cohen-Tannoudji, C., Diu, B. & Laloë, F. *Quantum mechanics*. (Wiley, 1977).
20 Nguyen, X. T. *et al.* Ultrafast Charge Carrier Relaxation in Inorganic Halide Perovskite Single Crystals Probed by Two-Dimensional Electronic Spectroscopy. *The Journal of Physical Chemistry Letters* **10**, 5414-5421 (2019).
21 De Sio, A., Nguyen, X. T. & Lienau, C. Signatures of Strong Vibronic Coupling Mediating Coherent Charge Transfer in Two-Dimensional Electronic Spectroscopy. *Zeitschrift für Naturforschung A* **74**, 721-737 (2019).
22 Palmieri, B., Abramavicius, D. & Mukamel, S. Lindblad equations for strongly coupled populations and coherences in photosynthetic complexes. *The Journal of Chemical Physics* **130**, 204512 (2009).
23 Blancon, J. C. *et al.* Scaling law for excitons in 2D perovskite quantum wells. *Nature Communications* **9**, 2254 (2018).
24 Menahem, M. *et al.* Strongly Anharmonic Octahedral Tilting in Two-Dimensional Hybrid Halide Perovskites. *ACS Nano* **15**, 10153-10162 (2021).
